# Modeling Honey Bee Populations

**DOI:** 10.1371/journal.pone.0130966

**Published:** 2015-07-06

**Authors:** David J. Torres, Ulises M. Ricoy, Shanae Roybal

**Affiliations:** 1 Department of Mathematics and Physical Science, Northern New Mexico College, Espanola, NM, USA; 2 Department of Biology, Northern New Mexico College, Espanola, NM, USA; University of North Carolina, Greensboro, UNITED STATES

## Abstract

Eusocial honey bee populations (*Apis mellifera*) employ an age stratification organization of egg, larvae, pupae, hive bees and foraging bees. Understanding the recent decline in honey bee colonies hinges on understanding the factors that impact each of these different age castes. We first perform an analysis of steady state bee populations given mortality rates within each bee caste and find that the honey bee colony is highly susceptible to hive and pupae mortality rates. Subsequently, we study transient bee population dynamics by building upon the modeling foundation established by Schmickl and Crailsheim and Khoury et al. Our transient model based on differential equations accounts for the effects of pheromones in slowing the maturation of hive bees to foraging bees, the increased mortality of larvae in the absence of sufficient hive bees, and the effects of food scarcity. We also conduct sensitivity studies and show the effects of parameter variations on the colony population.

## Introduction

Globally, 87 of the most prominent 115 food crops rely on animal pollination. Honey bees contribute more than 15 billion dollars to the US economy through their vital role in pollinating fruits, nuts and vegetables [1]. US honey bee colonies have experienced a steady decline from 6 million colonies in 1947 to 2.5 million today. Recently the declines have been even more acute. With the exception of the 2013–14 overwintering loss, beekeepers have experienced an average loss of 30% since 2006 compared to the historical rate of 10–15% [1] and a portion of the losses are attributable to a new syndrome called Colony Collapse Disorder. Recent winter losses in Europe have a ranged from 3.5% to 33.6% [2]. Many agree that the decline of honey bees (*Apis mellifera*) is due to many factors which include the *Varroa destructor* mite, bee viruses, the microsporidian protozoa *Nosema ceranae*, pesticides, environmental stresses, and bee management practices [3–5]. Due to the expense and difficulty in studying each of these stresses separately or in combination in the field, researchers have designed mathematical models of bee populations.

A useful review of honey bee models can be found in Becher et al. [6]. The authors sort models into three main categories: “colony models” which model in-hive dynamics, “varroa models” which model the interaction between bees and mites, and “foraging models” which model the efficiency of foraging in diverse time-dependent landscapes. Our model falls into the colony model category since we track each day in the life of a bee as it progresses from an egg to a larvae, pupae, hive bee and finally to a foraging bee. We believe that each day in a life of a bee should be tracked to account for time lags between influences of one caste on another. Other colony models and their descriptions are discussed below.

DeGrandi-Hoffman et al. [7] construct a honey bee model (BEEPOP) whose components consist of the number of eggs laid by the queen and the proportion of eggs that develop into drone and female bees. Eggs are tracked daily as they age from larvae to adults. Foraging is limited to suitable temperatures, wind velocities and rainfall.

Martin [8] constructs a model of viral infection using mites as vectors. The model adapts the model established by DeGrandi-Hoffman and integrates meteorological conditions. Survival rates used are based on data from Fukuda and Sakagami [9].

Khoury et al. [10, 11] construct a model of differential equations which track the brood (egg, larvae and pupae), hive and foraging bee population as well as the amount of food. The egg laying rate of the queen decreases when food is scarce and when the hive population diminishes. The evolution of the hive bee population is modified by a recruitment term which accounts for the effect of the pheromone ethyl oleate. Ethyl oleate is produced by the foraging population and slows down the rate at which hive bees mature into foraging bees. Bees are removed from the population as foragers through a term which models the death rate.

Russell et al. [12] add seasonal effects to Khoury’s model by temporally modifying the egg laying rate of the queen bee, and the collection and the death rate of the foraging bee. The maximum queen laying rate is based on a correlation which is a function of the hive and forager population.

Schmickl and Crailsheim [13] provide one of the most detailed **ho**ney bee **po**pulation **mo**dels (HoPoMo) of honey bee dynamics consisting of 60 equations that track every day in the life of a bee before it reaches adulthood. The egg laying rate of the queen is modeled using a seasonal correlation. They also require eggs to have sufficient space in the honeycomb. Eggs, larvae and pupae age and die according to caste dependent mortality rates. In addition, larvae are reduced in the absence of sufficient nurse bees and larvae are cannibalized when pollen stores are low. Adult bees are partitioned and prioritized according to colony tasks. These tasks include nursing, pollen foraging, nectar processing and nectar foraging. Nursing and pollen foraging carry the highest priority in the bee colony. Pollen, nectar and honey stores are also tracked. Comparisons are made between model predictions and experimental data and sensitivity analyses are performed.

Becher et al. [14] construct an extensive model (BEEHAVE) based on their literature review of all available models. The model incorporates colony dynamics, the effects of the varroa mite and viruses, and a foraging model which integrates data from the landscape and its flowering plants. The colony component of the model is based on age castes (eggs, larvae, pupae and adults) and properties within each caste (gender, infection with varroa mites or viruses, and mortality) determine the survival rates.

We design a steady state and a transient model of honey bee dynamics. In the steady state model, we calculate the long term behavior of bee populations in the absence of seasonal variations using geometric series. While the colony has many positive and negative feedback loops controlling bee maturation mediated by a large number of pheromones and mechanical processes, we focus on two specific pheromones. Our model accounts for brood pheromone and ethyl oleate pheromone by slowing (or in their absence accelerating) the maturation of hive bees into foraging bees [15, 16]. We also increase the mortality of larvae in the absence of sufficient nursing hive bees. The steady state model is useful in that it allows us to assess the effect of increased mortality in a specific bee caste and the effects of these pheromones on bee survival.

Our transient model most closely matches the model of Schmickl and Crailsheim [13]. However, our model is based on differential equations and continues to track each day in the adult life of the bee whereas Schmickl and Crailsheim cease to track daily populations after bees reach adulthood. While Schmickl and Crailsheim delegate and prioritize the tasks of nursing, foraging, nectar processing and miscellaneous tasks to adults regardless of age, we continue to track day to day populations of bees in the hive and forager castes and delegate tasks to each age group. Our transient model also tracks a processing caste (composed of older hive bees) since according to Johnson [17, 18], the task repertoire of bees ranging in age from 12–21 days actually includes “15 tasks ranging from nest building and maintenance, nectar receiving and processing, to guarding the nest entrance.” Seeley [19] also compiles a list of 15 tasks unrelated to brood care.

Our model and Schmickl and Crailsheim’s reduce the survival rate of larvae if the ratio of hive to larvae bees falls below a healthy ratio. We also reduce the survival rate of bees in the absence of sufficient food. We believe our model combines features of models from Schmickl and Crailsheim [13] and the differential equation formulation of Khoury et al. [10, 11] to form an original model based largely on parameters that can be measured experimentally. Sensitivity studies are performed with parameters that are difficult to estimate.

Section 1 describes the steady state model and illustrates how increased mortality in bee castes and pheromones affect the overall bee colony. Section 2 explains the transient model and the management of pheromones and food scarcity effects within the model. Results and sensitivity studies using the transient model are discussed in Section 3.

## 1 Steady State Model

If one assumes a constant egg laying rate per day *E*_0_, a daily survival rate within each bee caste *S*_*egg*_, *S*_*larvae*_, *S*_*pupae*_, *S*_*hive*_, *S*_*forager*_, and the number of days spent in each bee caste *n*_*egg*_, *n*_*larvae*_, *n*_*pupae*_, *n*_*hive*_, *n*_*forager*_, one can compute the steady state distribution of the number of bees within each caste (E: Eggs, L: Larvae, P: Pupae, H: Hive, F: Forager) using geometric series,

$$\begin{array}{c} E=E_{0}\sum_{i=0}^{n_{egg}-1}S_{egg}^{i}=E_{0}\frac{1-S_{egg}^{n_{egg}}}{1-S_{egg}}, \end{array}$$ (1)

$$\begin{array}{ccc} L & = & E_{0}S_{egg}^{n_{egg}}\sum_{i=0}^{n_{larvae}-1}S_{larvae}^{i}=\\ & & \;E_{0}S_{egg}^{n_{egg}}\frac{1-S_{larvae}^{n_{larvae}}}{1-S_{larvae}}, \end{array}$$ (2)

$$\begin{array}{ccc} P & = & E_{0}S_{egg}^{n_{egg}}S_{larvae}^{n_{larvae}}\sum_{i=0}^{n_{pupae}-1}S_{pupae}^{i}=\\ & & \;E_{0}S_{egg}^{n_{egg}}S_{larvae}^{n_{larvae}}\frac{1-S_{pupae}^{n_{pupae}}}{1-S_{pupae}}, \end{array}$$ (3)

$$\begin{array}{ccc} H & = & E_{0}S_{egg}^{n_{egg}}S_{larvae}^{n_{larvae}}S_{pupae}^{n_{pupae}}\sum_{i=0}^{n_{hive}-1}S_{hive}^{i}=\\ & & \;E_{0}S_{egg}^{n_{egg}}S_{larvae}^{n_{larvae}}S_{pupae}^{n_{pupae}}\frac{1-S_{hive}^{n_{hive}}}{1-S_{hive}}, \end{array}$$ (4)

$$\begin{array}{ccc} F & = & E_{0}S^{*}\sum_{i=0}^{n_{forager}-1}S_{forager}^{i}\\ & = & E_{0}S^{*}\frac{1-S_{forager}^{n_{forager}}}{1-S_{forager}} \end{array}$$ (5)

where $S^{*}=S_{egg}^{n_{egg}}S_{larvae}^{n_{larvae}}S_{pupae}^{n_{pupae}}S_{hive}^{n_{hive}}.$ In these equations, we lump the drones with the hive bees since they represent a small proportion of the hive caste. Schmickl and Crailsheim [13] similarly characterize the natural survival rates of bees within each caste in their HoPoMo model. Table 1 summarizes the variables used in the steady state model as well as the transient model. The total number of bees *T* in the colony (brood + adult) can be found by summing up the bees from each caste

$$\begin{array}{c} T=E+L+P+H+F. \end{array}$$ (6)

The number of days spent in each bee caste are based on values provided by Schmickl et al. [13] and are listed in Table 2. Daily mortality rates are chosen based on rates used by Schmickl et al. [13] who base their rates on experimental data provided by Sakagami and Fukuda [20]. Note that daily mortality rates *m* can be converted to daily survival rates *S* using the equation, *S* = 1 − *m*. We also take data from Fukuda and Sakagami [9] to establish a second source of survival rates. Daily survival rates in Table 3 based on [13] are listed in row I and survival rates based on [9] are listed in row II. The forager survival rate (.9) in row II comes from Russell et al. [12]. The survival rate of .985 for the mortality of hive bees is a rate that can be deduced from the observations of Harbo [21] (after the class survival rate of .87 is converted to a daily rate). Note that there is a sharp increase in the mortality when transitioning to foraging bees. This is accepted by many authors including [20] and [22]. For comparison, row III assumes there is a 100% survival rate within all castes.

**Table 1:** Variables used in honey bee model.

$a_{i}^{e},a_{i}^{b},a_{i}$	Maturation terms to account for pheromones
*B*_0_	Seasonally adjusted egg laying rate of queen
*B*_*i*_	Number of bees that are *i* days old
*C*_*egg*_, *C*_*larvae*_, *C*_*pupae*_, *C*_*hive*_, *C*_*forager*_	Daily consumption rates of bee class in grams
*d*_*i*_	Larvae deaths per day
*D*	Food deficit quantity
*E*	Number of eggs
*E*_0_	Daily egg laying rate of queen bee
*f*	Food reserves in the colony (grams)
*f*_*d*_	Daily food requirement of colony (grams)
*f*_*a*_, *f*_*i*_	Available food (grams), Inaccessible food (grams)
*f*_*L*_, *f*_*H*_, *f*_*F*_	Daily food requirement for larvae, hive and forager castes (grams)
*F*	Number of foraging bees
*h* ^*e*^, *h* ^*b*^	Terms used in computing $a_{i}^{e}$ and $a_{i}^{b}$
*H*	Number of hive bees
*i*	Denotes the age of bee in days
*L*	Number of larvae
*n*_*egg*_, *n*_*larvae*_, *n*_*pupae*_, *n*_*hive*_, *n*_*forager*_	Number of days spent within each bee class
*n*_*a*_	Parameter used to determine how many days are subject to acceleration
*N*	Number of nursing bees
*p*	Number of grams of pollen collected by one foraging bee in one day
*P*	Number of pupae
*Q*	Number of processors
*r*	$(R_{L}^{H})/{\left(R_{L}^{H}\right)}_{healthy}$ or $(R_{L}^{N})/{\left(R_{L}^{N}\right)}_{healthy}$
$R_{L}^{H}$, ${\left(R_{L}^{H}\right)}_{healthy}$	Ratio and healthy ratio of number of hive bees to larvae
$R_{L}^{N}$, ${\left(R_{L}^{N}\right)}_{healthy}$	Ratio and healthy ratio of number of nursing bees to larvae
$R_{F}^{H}$, ${\left(R_{F}^{H}\right)}_{healthy}$	Ratio and healthy ratio of number of hive to foraging bees
$R_{F}^{Q}$, ${\left(R_{F}^{Q}\right)}_{healthy}$	Ratio and healthy ratio of number of processors to foraging bees
*S*_*egg*_, *S*_*larvae*_, *S*_*pupae*_, *S*_*hive*_, *S*_*forager*_	Survival rate within each bee caste in steady state model
*S*_*i*_	Survival rate of bee classes in temporal model
*S*_*L*_, *S*_*H*_, *S*_*F*_	Factors that reduce survival rate due to food scarcity
$S_{larvae}^{reduce}$	Reduced larvae survival rate due to insufficient hive bees
$s(t),\tilde{s}(t)$	Seasonal effect which modifies egg laying rate and foraging rate *p*
*t*	Time (in units of days)
*T*	Total number of bees in colony T = E + L + P + H + F
*w*_*i*_	Weight of larvae that are *i* days old
*x*_1_, *x*_2_, *x*_3_, *x*_4_, *x*_5_	Parameters used in computing seasonal terms $s(t),\tilde{s}(t)$
△*t*	Time step
*γ*_*L*_	Factor which determines nutritional value of cannibalized larvae
*ξ*	Factor to promote early maturation of hive bees during food scarcity

**Table 2:** Days in bee class.

*n*_*egg*_	*n*_*larvae*_	*n*_*pupae*_	*n*_*hive*_	*n*_*forager*_
3	5	12	21	14

**Table 3:** Survival rates.

	*S*_*egg*_	*S*_*larvae*_	*S*_*pupae*_	*S*_*hive*_	*S*_*forager*_
I	.97	.99	.999	.985	.955
II	.94	.917	.985	.985	.9
III	1.0	1.0	1.0	1.0	1.0

Table 4 shows the maximum colony size and percentage of the population in each class with the three different survival rates (S) (I, II and III) listed in Table 3 with an egg laying rate of *E*_0_ = 1500. The column T refers to the total number of bees (adult + brood). We note that the number of days spent as a hive bee and a forager is variable due to two pheromones: brood pheromone and ethyl oleate. Brood pheromone is produced by larvae and ethyl oleate is produced by foragers. Both delay the maturation of hive bees into foraging bees. Conversely colony food shortage accelerates the maturation of hive bees into foraging bees [23]. Seasonal variations can also affect the number of days spent within each caste. The lifespan of a worker bee (including time spent as a forager) can range from 15–38 days in the summer, 30–60 days in the spring and fall, and 150–200 days in the winter in the absence of foraging [24–27]. Rueppell et al. [28] observe that the number of days spent as a hive bee ranges from 8 to 42 days with a mean of 20.7 and the number of days spent as a foraging bee ranges from 1 to 42 days with a mean of 7.4 in May to July in Tempe, Arizona. The number of days spent as a hive and foraging bee in our steady state model in Table 2 are assumed to be averages in nonwinter seasons.

**Table 4:** Steady state bee colony caste percentage of total population T.

S	E	L	P	H	F	T
I	7.3	11.2	26.0	39.0	16.5	60,000
II	12.3	15.3	26.0	35.5	11	34,000
III	5.5	9.1	21.8	38.2	25.5	82,000

For comparison, Table 5 shows the percentages of the total population observed by Fukuda [29] in different bee castes as well as the total number of bees. We sample and average data at three times in the graph provided in [29] during the phase where the total populations stabilize. Note that the experimental data in Table 5 bear similarities to the survival rate I in Table 4 except for the total number of bees which we attribute primarily to differences in the egg laying rate.

**Table 5:** Percentages of bees in castes and total number of bees (T) according to Fukuda [29].

E	L	P	H	F	T
7.4	12.5	25.3	37.9	16.9	49,500

In our steady state model, we also track whether foragers bring in sufficient food to sustain the colony. If we assume each forager brings in *p* grams of food (nectar + pollen) per day, the following inequality must hold in order for the colony to be ultimately self-sustaining,

$$\begin{array}{c} Fp\geq LC_{larvae}+HC_{hive}+FC_{forager} \end{array}$$ (7)

where *C*_*larvae*_, *C*_*hive*_ and *C*_*forager*_ are the individual daily consumption rates in grams for larvae, hive bees and foraging bees. Food consumption rates of bees are based on data provided by Khoury et al. [11] and are listed in Table 6. We also assume p = .1 gram/(bee ⋅ day) based on estimates by Russell et al. [12]. Obviously, if food reserves are present, Eq (7) is not applicable. However, a steady state model is justified in using (7) because over the long term, all food reserves will be exhausted.

**Table 6:** Consumption rates [grams/(bee ⋅ day)].

*C*_*egg*_	*C*_*larvae*_	*C*_*pupae*_	*C*_*hive*_	*C*_*forager*_
0	.018	0	.007	.007

Finally an adequate larvae bee to nurse ratio must be maintained. Nurse bees are young hive bees responsible for feeding larvae which require a constant supply of proteins and carbohydrates [30]. Larvae are first fed nurse hive bee jelly that is produced by their hypopharyngeal glands followed by a combination of nectar and predigested protein-rich pollen [31, 32]. Consequently our model assumes the larvae survival rate will decrease if the colony lacks sufficient nurse bees. Define the hive to larvae ratio

$$\begin{array}{c} R_{L}^{H}\equiv \frac{H}{L}. \end{array}$$ (8)

Furthermore define ${\left(R_{L}^{H}\right)}_{healthy}$ to be a healthy ratio. Schmickl et al. [13] use a value of ${\left(R_{L}^{H}\right)}_{healthy}=2$, based on data from Eischen et al. [33]. We assume the ratio accounts for the actual portion of the hive bee caste that are nursing bees. Older hive bees are also responsible for honeycomb construction which house larvae. The survival of the larvae is reduced by *r* ^*α*^

$$\begin{array}{c} S_{larvae}^{reduce}=S_{larvae}r^{\alpha } \end{array}$$ (9)

when $R_{L}^{H}<{\left(R_{L}^{H}\right)}_{healthy}$ where

$$\begin{array}{c} r=\frac{R_{L}^{H}}{{\left(R_{L}^{H}\right)}_{healthy}}. \end{array}$$ (10)

**Fig 1:**
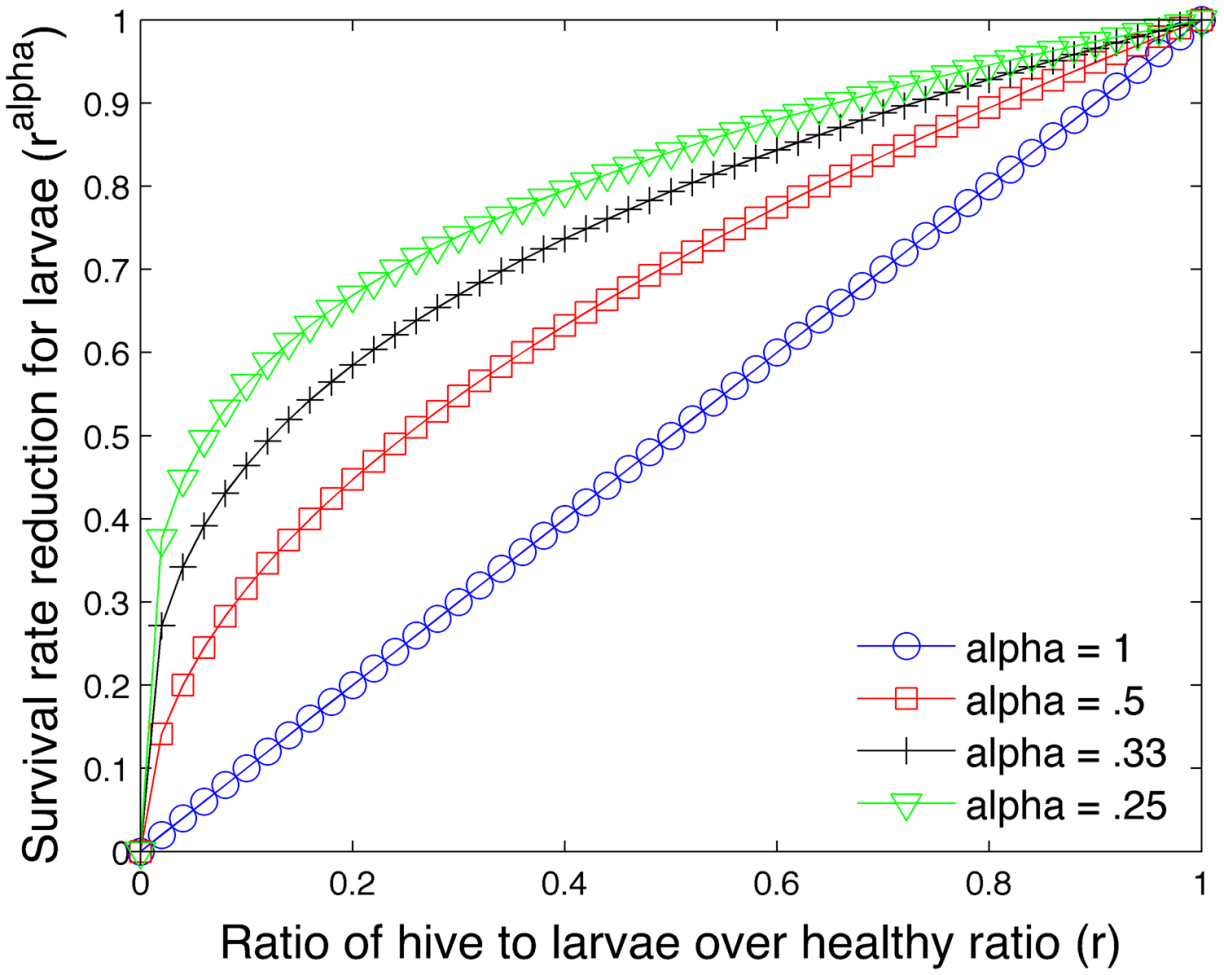
Effect of exponent *α* on survival rate of larvae.

An exponent *α* < 1 minimizes the impact of reduced nurses. We use *α* = .25. Fig 1 plots *r* ^*α*^ versus *r* for different values of *α*.

Khoury et al. [11] and Schmickl et al. [13] also reduce the amount of brood and larvae respectively in the absence of sufficient hive bees. Khoury et al. use the factor $\frac{H}{H+\nu }$ and the parameter *ν* to reduce the egg laying rate of the queen in the evolution equation for brood. Schmickl et al. reduce the survival rate of the larvae through a nursing quality factor and prescribe a minimum value greater than zero for the larvae survival rate.

According to Castillo et al. [15], the physiological change from hive to forager bees is delayed by the pheromone ethyl oleate which is manufactured by foragers. Ethyl oleate also helps maintain the beneficial ratio of nurse to forager bees [34]. We account for the pheromone ethyl oleate in the steady state model by reducing or increasing the length of time spent as a hive bee. The amount of days added or subtracted to the hive bee class (*n*_*hive*_) is computed to make the ratio of hive bees to foragers $R_{F}^{H}=\frac{H}{F}$ as close as possible to a healthy ratio,

$$\begin{array}{c} R_{F}^{H}\approx {\left(R_{F}^{H}\right)}_{healthy}. \end{array}$$ (11)

We assume ${\left(R_{F}^{H}\right)}_{healthy}$ to be 2.3 based on survival rate I in Table 4 and Table 5. In addition, the healthy ratio is reduced by .5 in the absence of sufficient food to encourage the creation of additional foragers.

Brood pheromone is a mixture of fatty acid esters found on the surface of larvae [16]. It serves to communicate the presence of larvae and functions in much the same way as ethyl oleate by slowing down the maturation of hive bees. According to Sagili et al. [16], the age of first foraging decreased in low brood pheromone treated colonies. Mathematically, brood pheromone is managed in the same way as ethyl oleate. The amount of days added or subtracted to the hive bee class (*n*_*hive*_) is computed to make the ratio of hive bees to larvae as close as possible to a healthy ratio,

$$\begin{array}{c} R_{L}^{H}\approx {\left(R_{L}^{H}\right)}_{healthy}\approx 2. \end{array}$$ (12)

In the steady state model, we use either the brood pheromone module or the ethyl oleate module but not both simultaneously since both change the number of days spent as a hive bee *n*_*hive*_. A composite model would need to prioritize which pheromone takes precedence.

**Fig 2:**
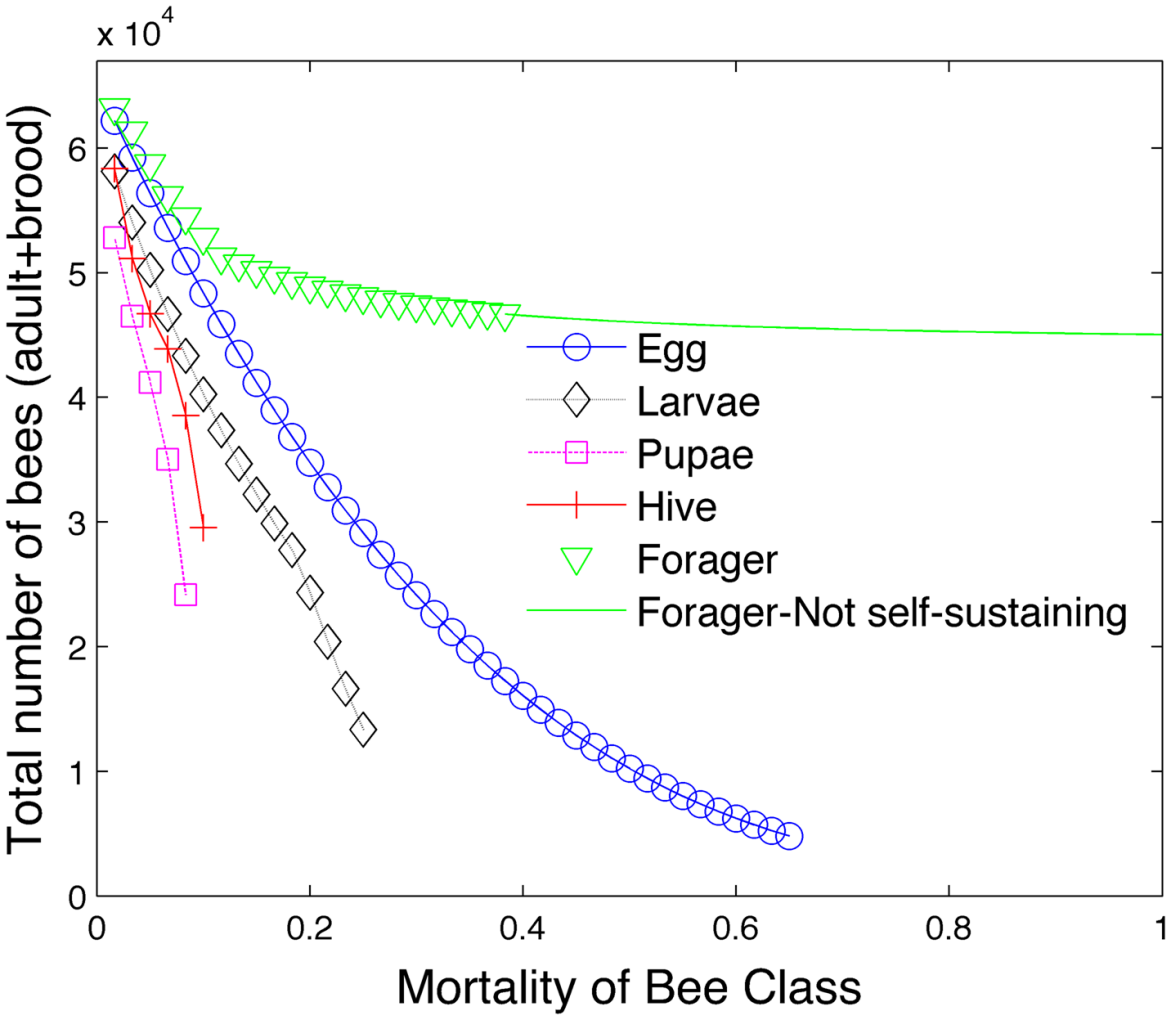
Effect of mortality on bee population.

Fig 2 shows the effect of varying mortality on the total bee population. In this figure, the mortality rates of all bee classes are based on survival rate I shown in Table 3 except for one bee caste. The mortality rate of this one bee caste is progressively increased until the colony collapses. We apply (9) to modify the survival rate of larvae in the absence of sufficient hive bees. Eq (11) is used to modify the number of days spent as a hive bee if the ratio of hive bees to foragers deviates from a healthy ratio. The brood pheromone Eq (12) is not used in this simulation. Colony collapse is assumed to occur when the hive bee population falls below 1000 which we assume to be a colony size that is not viable. A solid line is used to represent a honey bee colony that is viable but not self-sustaining in terms of food requirements.

To clarify what is being plotted, let us consider the green triangles. These symbols represent the graph of the total bee population (egg + larvae + pupae + hive + forager) as the mortality of the forager class increases. Mortality rates of the other bee classes are determined by the rates in survival rate I and Eq (9). We see that the colony can still survive even with very high forager mortality rates. However, the green triangles eventually transition into the solid line. This is the point beyond which the colony is not self-sustaining in regards to food (i.e. Eq (7) is not satisfied). Similarly the blue circles represent the total bee population as the mortality of the egg class increases.

Fig 2 shows that the maximum colony size predicted by the steady state model is slightly higher than 60,000 bees using survival rate I. Since there are no solid lines for the egg, larvae, pupae and hive caste graphs, the figure shows that the bee colony is not viable even with unlimited food reserves beyond a specific mortality rate for these bee classes. We also note that even small mortality rates in the pupae, hive and larvae population have a devastating effect on the colony size. While the colony is still sensitive to mortality rates in the egg populations, it is slightly more resilient to mortality rates in this caste compared to the hive and pupae castes.

**Fig 3:**
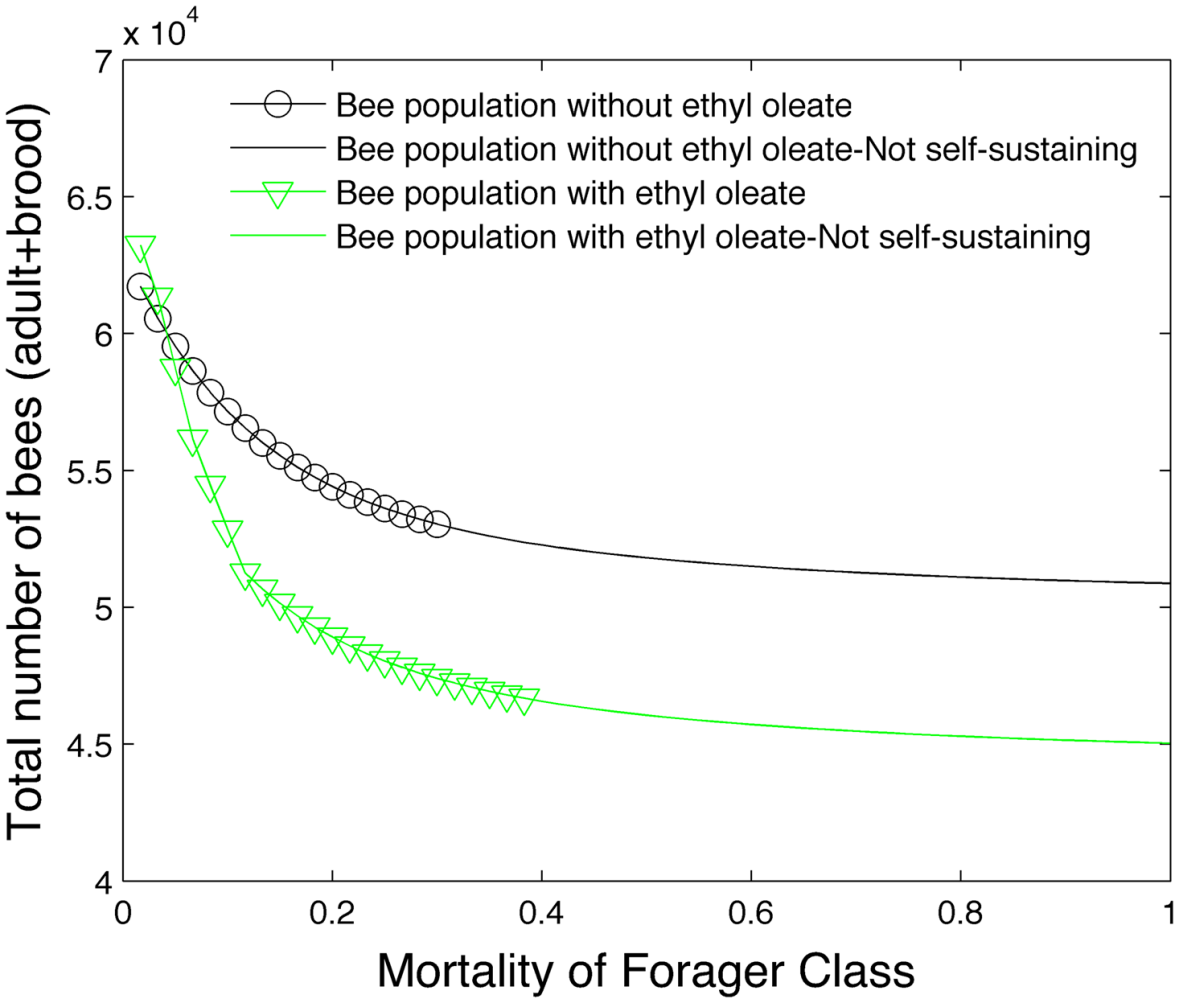
Effect of ethyl oleate on bee population.

Fig 3 demonstrates the effect of the pheromone ethyl oleate in the model. The total bee population is plotted on the vertical axis and the forager mortality is plotted on the horizontal axis under two conditions. In the first condition, the number of days spent as a hive and forager bee is fixed and the effects of ethyl oleate are excluded (black circles). In the second condition, the number of days spent as a hive and forager bee is variable and the effects of ethyl oleate are included (green triangles). We see that while ethyl oleate reduces the total bee population, it does allow the bee colony to be self-sustaining under higher forager mortality rates. The effect of brood pheromone was turned off in this particular simulation to isolate the effect of ethyl oleate.

**Fig 4:**
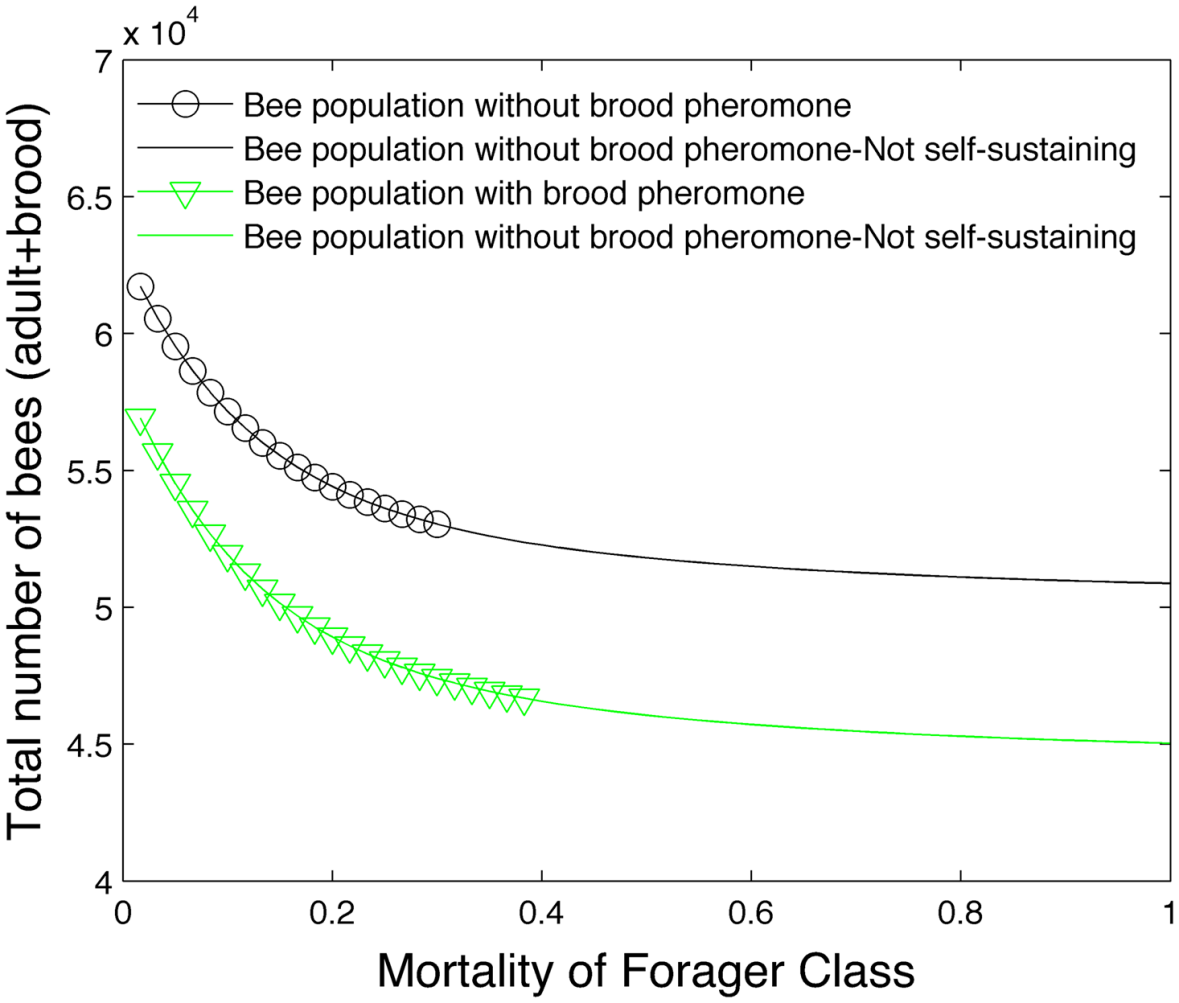
Effect of brood pheromone on bee population.

Fig 4 demonstrates the effect of brood pheromone in the model. In the first condition, the number of days spent as a hive and forager bee is fixed and the effects of brood pheromone are excluded (black circles). In the second condition, the number of days spent as a hive and forager bee is variable and the effects of brood pheromone are included (green triangles). Again we see that while brood pheromone reduces the total bee population, it does allow the bee colony to be self-sustaining under higher forager mortality rates. The effect of ethyl oleate was turned off in this particular simulation to isolate the effect of brood pheromone.

**Fig 5:**
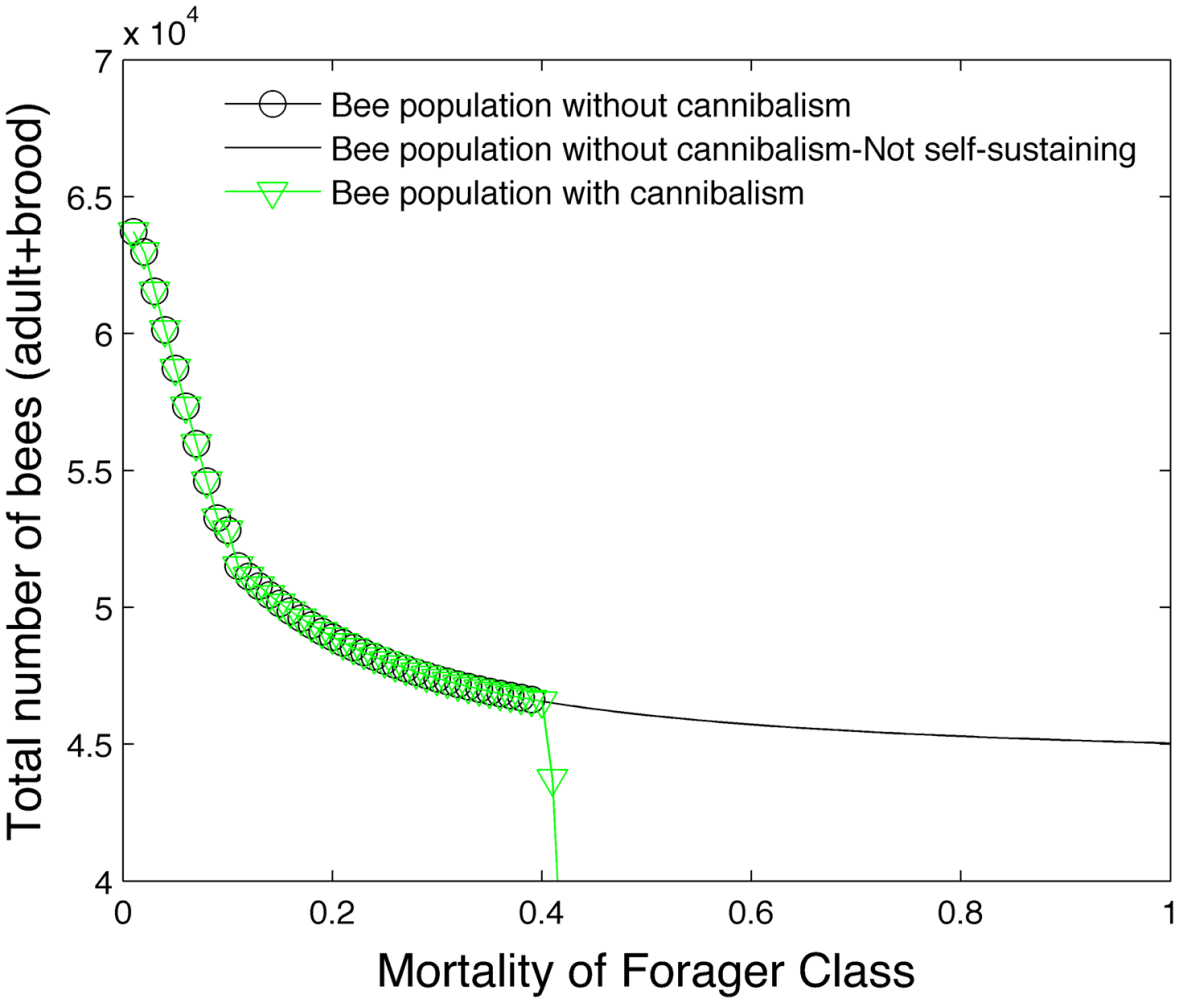
Effect of cannibalism on bee population.

Fig 5 demonstrates the effect of cannibalism in the model which uses the ethyl oleate pheromone. All previous steady state simulations did not incorporate cannibalism. In the first condition (black circles and line), cannibalism is excluded from the model. In the second condition (green triangles), larvae are cannibalized in the absence of sufficient food to make the colony self-sustaining. The nutritional value of a larvae is assigned to be equal to an average experimental weight (50 mg) times one-half. The number of cannibalized larvae is computed to offset any food deficit. Our model shows that cannibalism precipitates the rapid collapse of the colony. Any food benefit gained from the cannibalized larvae is offset by the eventual lack of hive bees to care for larvae and the shortage of foragers to bring in food. Perhaps the evolutionary advantage of cannibalism is limited to short transient intervals which can only be captured in a transient model. See Section 3.1.5.

In the absence of seasonal effects, the steady state model is a useful tool in obtaining final numbers of bees in the colony (assuming a constant egg laying rate) and isolates the effect of a single variable (e.g. caste mortality, pheromones and cannibalism). Although it neglects seasonal variations in foraging and egg laying, these simplifying assumptions allow the steady state model to predict final numbers and ratios of bee castes under different mortality and pheromone scenarios.

## 2 Transient Model

While the steady state model predicts the long term behavior of the bee colony, it does not capture time fluctuations that occur in the colony and seasonal variations. Hence prudence requires the development of a time dependent or transient colony model. Currently our transient model does not consider infestations nor does it account for spatially and temporally varying foraging landscapes. However these effects could potentially be accounted for by varying the mortality rate of bee castes as well as the foraging rate.

We begin with our fundamental equation

$$\begin{array}{c} \frac{dB_{i}}{dt}=\left(S_{i-1}B_{i-1}-B_{i}\right)a_{i},\;\;\;1\leq i\leq 55 \end{array}$$ (13)

where *i* refers to the age in days, *S*_*i*_ is the daily survival rate of a bee that is *i* days old, *B*_*i*_ is the number of bees that are *i* days old,

$$\begin{array}{c} B_{0}=s\left(t\right)E_{0} \end{array}$$ (14)

represents the seasonally adjusted daily egg laying rate of the queen (invoked when *i* = 1 in (13)), and *a*_*i*_ is an acceleration term which accelerates or decelerates the maturation of hive bees due to the presence of pheromones. Eq (13) is actually 55 separate coupled equations which can be solved analytically when the egg laying rate *E*_0_ and survival rates *S*_*i*_ are not functions of time or bee caste populations and in the absence of seasonal and food scarcity effects and pheromones (*s*(*t*) = *a*_*i*_ = 1).

$$\begin{array}{c} B_{i}\left(t\right)=E_{0}\prod_{j=1}^{j=i-1}S_{j}+\sum_{k=0}^{i-1}[(\prod_{j=i-k}^{i-1}S_{j})(B_{i-k}\left(0\right)-E_{0}\prod_{j=1}^{i-k-1}S_{j})f_{k}\left(t\right)] \end{array}$$

where

$$\begin{array}{c} f_{k}\left(t\right)=\frac{t^{k}}{k!}e^{-t}, \end{array}$$

and the products $\prod_{j=b}^{t}$ are set to 1 if the lower bound *b* is greater than the upper bound *t*. In addition *S*_0_ is assumed to be 1. Allowing survival and acceleration rates to be themselves functions of bee caste populations introduces nonlinear effects and precludes an analytical solution.

Eq (13) states that in the absence of pheromones, the rate of change of bees that are *i* days old ($\frac{dB_{i}}{dt}$) is increased by aging bees that are *i* − 1 days old times a survival rate (*S*_*i*−1_ *B*_*i*−1_) and decreased through aging by the current number of bees *B*_*i*_. Table 7 shows how the number of eggs (E), larvae (L), pupae (P), nursing bees (N), processing bees (Q), hive bees (H), and foragers (F) are computed by summing over ranges of days (*i*). We use the same ranges of days in the transient model as the steady state model. Note also that we have added a nursing caste (N) and a processing caste (Q) to the transient model. The nursing and processing castes are subsets of the hive caste. All daily survival rates are based on the daily survival I rates provided in Table 3. In addition, the larvae survival rate *S*_*i*_, 4 ≤ *i* ≤ 8 is decreased in the absence of sufficient hive bees using Eq (9). However *r* is computed using only the nursing bees

$$\begin{array}{c} r=\frac{R_{L}^{N}}{{\left(R_{L}^{N}\right)}_{healthy}} \end{array}$$ (15)

if $R_{L}^{N}<{\left(R_{L}^{N}\right)}_{healthy}$ where ${\left(R_{L}^{N}\right)}_{healthy}=.5{\left(R_{L}^{H}\right)}_{healthy}=1.$ If $R_{L}^{N}\geq {\left(R_{L}^{N}\right)}_{healthy}$, *r* = 1.

**Table 7:** Day ranges used to calculate bee demographics.

Sum over *B*_*i*_	Bee class
$\sum_{i=1}^{i=3}B_{i}$	Egg (E)
$\sum_{i=4}^{i=8}B_{i}$	Larvae (L)
$\sum_{i=9}^{i=20}B_{i}$	Pupae (P)
$\sum_{i=21}^{i=30}B_{i}$	Nursing (N)
$\sum_{i=31}^{i=41}B_{i}$	Processing (Q)
$\sum_{i=21}^{i=41}B_{i}$	Hive (H)
$\sum_{i=42}^{i=55}B_{i}$	Forager (F)

### 2.1 Processing caste

We track the number of bees in the processing caste (*Q*) by summing all bees ranging from 31 days to 41 days old,

$$\begin{array}{c} Q=\sum_{i=31}^{41}B_{i}. \end{array}$$ (16)

Processors are responsible for tasks unrelated to nursing (e.g. food processing and nest building). To account for the effects of reduced processors, we determine a healthy ratio of processors to foragers ${\left(R_{F}^{Q}\right)}_{healthy}$. If the actual ratio of processors to foragers

$$\begin{array}{c} R_{F}^{Q}=\frac{Q}{F} \end{array}$$ (17)

is less than the healthy ratio $R_{F}^{Q}<{\left(R_{F}^{Q}\right)}_{healthy}=.8$, we reduce the food (pollen and nectar) foraging rate *p* by the factor

$$\begin{array}{c} f_{Q}=\frac{R_{F}^{Q}}{{\left(R_{F}^{Q}\right)}_{healthy}}. \end{array}$$ (18)

### 2.2 Effects of food scarcity

Bee deaths due to insufficient food are computed by first tracking the amount of food *f* in the colony using the differential equation

$$\begin{array}{c} \frac{df}{dt}=s\left(t\right)\left(f_{Q}\right)pF+\gamma_{L}\sum_{i=4}^{i=8}w_{i}d_{i}-f_{d},\;\;f\geq 0 \end{array}$$ (19)

where *f*_*d*_ represents the daily food requirement,

$$\begin{array}{c} f_{d}=f_{L}+f_{H}+f_{F} \end{array}$$ (20)

and *f*_*L*_ = *LC*_*larvae*_, *f*_*H*_ = *HC*_*hive*_ and *f*_*F*_ = *FC*_*forager*_ represent the daily food requirements of larvae, hive, and forager bees respectively. Since the factors *C*_*larvae*_, *C*_*hive*_ and *C*_*forager*_ represent the consumption rates per day per bee for larvae, hive and foraging bees, it follows that *LC*_*larvae*_, *HC*_*hive*_ and *FC*_*forager*_ represent the food consumed by the entire larvae, hive and forager populations in a day. Section 2.1 describes *f*_*Q*_ ≤ 1 which reduces the foraging rate in the absence of sufficient processing bees, and *s*(*t*) accounts for seasonal effects that affect the foraging rate *p*. The term $\gamma_{L}\sum_{i=4}^{i=8}w_{i}d_{i}$ refers to the daily nutritional value gained from cannibalizing the larvae population when food is scarce. In addition, *w*_*i*_ represents the average weight of larvae at day *i*, 4 ≤ *i* ≤ 8, and *d*_*i*_ represents the number of larvae deaths per day when the colony does not have sufficient food. The weights *w*_*i*_ are obtained from Schmickl and Crailsheim [13] who use data from Stabe [35] and Wang [36], *w*_*i*_ = {.1 mg,.6 mg, 20 mg, 80 mg, 150 mg}.

The colony only experiences increased mortality due to insufficient food if *f*_*a*_ < *f*_*d*_ where *f*_*a*_ represents the accessible food,

$$\begin{array}{c} f_{a}=max\left\{f-f_{i},0\right\} \end{array}$$ (21)

and *f*_*i*_ the amount of inaccessible food (set to 100 grams). We also define the food deficit *D* to be

$$\begin{array}{c} D=\{\begin{array}{cc} |f_{a}-f_{d}| & f_{a}-f_{d}<0\\ 0 & f_{a}-f_{d}\geq 0 \end{array}. \end{array}$$

We set *γ*_*L*_ to .5 when *D* > 0 which assumes the nutritional value gained from cannibalized larvae is half their weight. If the accessible food *f*_*a*_ present is greater than the food requirement *f*_*d*_, *D* = 0, and the colony experiences no food deaths. The parameter *γ*_*L*_ is also set to zero. To compute the increased mortality when *D* > 0, we use the following conditions

$$\begin{array}{c} \begin{array}{ccc} \left(1\right) & S_{L}=max\left\{\frac{f_{a}-\left(f_{H}+f_{F}\right)}{f_{L}},S_{min,L}\right\},S_{H}=1,S_{F}=1 & if\;\;f_{H}+f_{F}\leq f_{a}<f_{d}\\ \left(2\right) & S_{L}=S_{min,L},S_{H}=max\left\{\frac{f_{a}}{f_{H}+f_{F}},S_{min,H}\right\},S_{F}=max\left\{\frac{f_{a}}{f_{H}+f_{F}},S_{min,F}\right\} & if\;\;0\leq f_{a}<f_{H}+f_{F}\\ \left(3\right) & S_{L}=S_{min,L},S_{H}=S_{min,H},S_{F}=S_{min,F} & if\;\;f_{a}\leq 0 \end{array}. \end{array}$$

Condition (1) assumes that any available food will be consumed by hive and forager bees first. The remaining food *f*_*a*_ − (*f*_*H*_ + *f*_*H*_) is divided by the food need of the larvae *f*_*L*_ to compute a reduced survival rate *S*_*L*_ for the larvae caste. The hive and forager caste do not experience a reduced survival rate *S*_*H*_ = *S*_*F*_ = 1 since there is sufficient food to meet their needs. We also require *S*_*L*_ to be greater than *S*_*min*, *L*_ = .2.

Condition (2) assumes all available food will be used to feed the hive and forager caste. The larvae caste will experience the minimum survival rate *S*_*min*, *L*_. The reduced survival rate of hive and forager bees is computed by finding the ratio of available food *f*_*a*_ to the food need of hive and forager bees, *f*_*H*_ + *f*_*F*_. These reduced survival rates are limited by the minimal rates *S*_*min*, *H*_ = .5 and *S*_*min*, *F*_ = .67. Condition (3) sets all reduced survival rates to their minimum value if there is no available food.

The survival factors *S*_*L*_, *S*_*H*_ and *S*_*F*_ modify the survival rate in (13) by setting *S*_*i*_ equal to *S*_*i*_ *S*_*X*_ where *X* = {*L*, *H*, *F*} depending on the range of *i*’s.

### 2.3 Pheromones

We account for the effects of pheromones differently in the transient model than in the steady state model. The acceleration term *a*_*i*_ is normally set to one except for the older hive bees 41 − *n*_*a*_ ≤ *i* ≤ 41. It attempts to establish an ideal ratio of hive bees to foraging bees in the case of ethyl oleate and an ideal ratio of hive bees to larvae in the case of brood pheromone. Therefore *a*_*i*_ is set to be greater than one in the range 41 − *n*_*a*_ ≤ *i* ≤ 41 to accelerate the maturation of hive bees and less than one to decelerate their development into foragers.

The parameter *n*_*a*_ (which we set to 6) represents the number of days in the hive bee caste during which the maturation can be accelerated or decelerated. We calculate *a*_*i*_ using the formula

$$\begin{array}{c} a_{i}=a_{i}^{e}a_{i}^{b},\;\;41-n_{a}\leq i\leq 41 \end{array}$$ (22)

where $a_{i}^{e}$ accounts for ethyl oleate and $a_{i}^{b}$ accounts for brood pheromone. The computation of $a_{i}^{e}$ and $a_{i}^{b}$ is described in the next two subsections. Furthermore we require that

$$\begin{array}{c} \frac{1}{3}\leq a_{i}\leq 3,\;\;41-n_{a}\leq i\leq 41. \end{array}$$ (23)

We also set *a*_*i*_ = 1 for all *i*’s or days during the winter months.

#### 2.3.1 Ethyl oleate

In the case of ethyl oleate, an ideal ratio ${\left(R_{F}^{H}\right)}_{healthy}$ is determined and modified by a term $\xi \frac{D}{f_{d}}$ which accounts for food scarcity,

$$\begin{array}{c} {\left(R_{F}^{H}\right)}_{mod}={\left(R_{F}^{H}\right)}_{healthy}-\xi \frac{D}{f_{d}}. \end{array}$$ (24)

The term $\xi \frac{D}{f_{d}}$ encourages early maturation of hive bees in the absence of food by reducing the ideal ratio ${\left(R_{F}^{H}\right)}_{healthy}$. The parameter *ξ* which we set to .5 controls the magnitude of this effect. We currently set ${\left(R_{F}^{H}\right)}_{healthy}=2.3$ to the same value used in the steady state model.

The acceleration term $a_{i}^{e}$ is then computed using

$$\begin{array}{c} a_{i}^{e}=\{\begin{array}{cc} 1+h^{e} & 41-n_{a}\leq i\leq 41\\ 1 & i<41-n_{a},\;\;\text{or}\;\;i>41 \end{array} \end{array}$$ (25)

where

$$\begin{array}{c} h^{e}=\frac{R_{F}^{H}-{\left(R_{F}^{H}\right)}_{mod}}{{\left(R_{F}^{H}\right)}_{mod}}. \end{array}$$ (26)

If there are insufficient hive bees, $R_{F}^{H}<{\left(R_{F}^{H}\right)}_{mod}$, *h*_*e*_ < 0 and the maturation of older hive bees will be decelerated. If there are too many hive bees, $R_{F}^{H}>{\left(R_{F}^{H}\right)}_{mod}$, *h*_*e*_ > 0 and the maturation of older hive bees will be accelerated. We also require $\frac{1}{3}\leq a_{i}^{e}\leq 3,\;\;41-n_{a}\leq i\leq 41$.

#### 2.3.2 Brood pheromone

In the case of brood pheromone, an ideal ratio ${\left(R_{L}^{H}\right)}_{healthy}$ is determined. We currently set ${\left(R_{L}^{H}\right)}_{healthy}=2.0$ to the same value used in the steady state model. The acceleration term $a_{i}^{b}$ is then computed using

$$\begin{array}{c} a_{i}^{b}=\{\begin{array}{cc} 1+.5h^{b} & 41-n_{a}\leq i\leq 41\\ 1 & i<41-n_{a},\;\;\text{or}\;\;i>41 \end{array} \end{array}$$ (27)

where

$$\begin{array}{c} h^{b}=\frac{R_{L}^{H}-{\left(R_{L}^{H}\right)}_{healthy}}{{\left(R_{L}^{H}\right)}_{healthy}}. \end{array}$$ (28)

If there are two few hive bees compared to larvae, $R_{L}^{H}<{\left(R_{L}^{H}\right)}_{healthy}$, *h* ^*b*^ will be negative, $a_{i}^{b}$ will be less than one and the maturation of hive bees into foragers will be slowed. If there are two many hive bees compared to larvae, *h* ^*b*^ will be positive and the maturation of hive bees into foragers will be accelerated. The factor .5 in Eq (27) reduces the magnitude of the effect of brood pheromone relative to ethyl oleate. We also require $\frac{1}{2}\leq a_{i}^{b}\leq 2,\;\;41-n_{a}\leq i\leq 41$. We also note that ethyl oleate and brood pheromone can counteract each other if $a_{i}^{e}>1$ and $a_{i}^{b}<1$ or vice versa.

### 2.4 Solution method

We use the first order Euler’s method to solve the equations (13). In the method, the time derivative $\frac{dB_{i}}{dt}$ is approximated with the first order finite difference quotient,

$$\begin{array}{c} \frac{dB_{i}}{dt}\approx \frac{B_{i}^{n+1}-B_{i}^{n}}{△t}. \end{array}$$ (29)

If the time step △*t* is one day, $B_{i}^{n}$ represents the number of bees that are *i* days old on the *n*th day of the simulation. If the time step is less than a day, $B_{i}^{n}$ represents the number bees that are *i* days old at time *n*△*t*. Substituting (29) into (13), we form the equation

$$\begin{array}{c} B_{i}^{n+1}=B_{i}^{n}+△t\left[\left(S_{i-1}B_{i-1}^{n}-B_{i}^{n}\right)a_{i}\right] \end{array}$$ (30)

Similarly Eq (19) can be transformed into

$$\begin{array}{c} f^{n+1}=f^{n}+△t(s\left(t^{n}\right)\left(f_{Q}\right)pF^{n}+\gamma_{L}\sum_{i=4}^{i=8}w_{i}d_{i}^{n}-f_{d}^{n}). \end{array}$$ (31)

Equations (30, 31) allow one to predict the population and food at time (*n* + 1)△*t* given the population and food at time *n*△*t*. They are implemented in a 500+ line MATLAB code. One can think of △*t* as the interval between snapshots of the honey bee colony. The accuracy of Euler’s method or any stable numerical method will improve as △*t* decreases. The next subsection attempts to answer how small △*t* should be to produce an accurate solution.

### 2.5 Convergence

**Fig 6:**
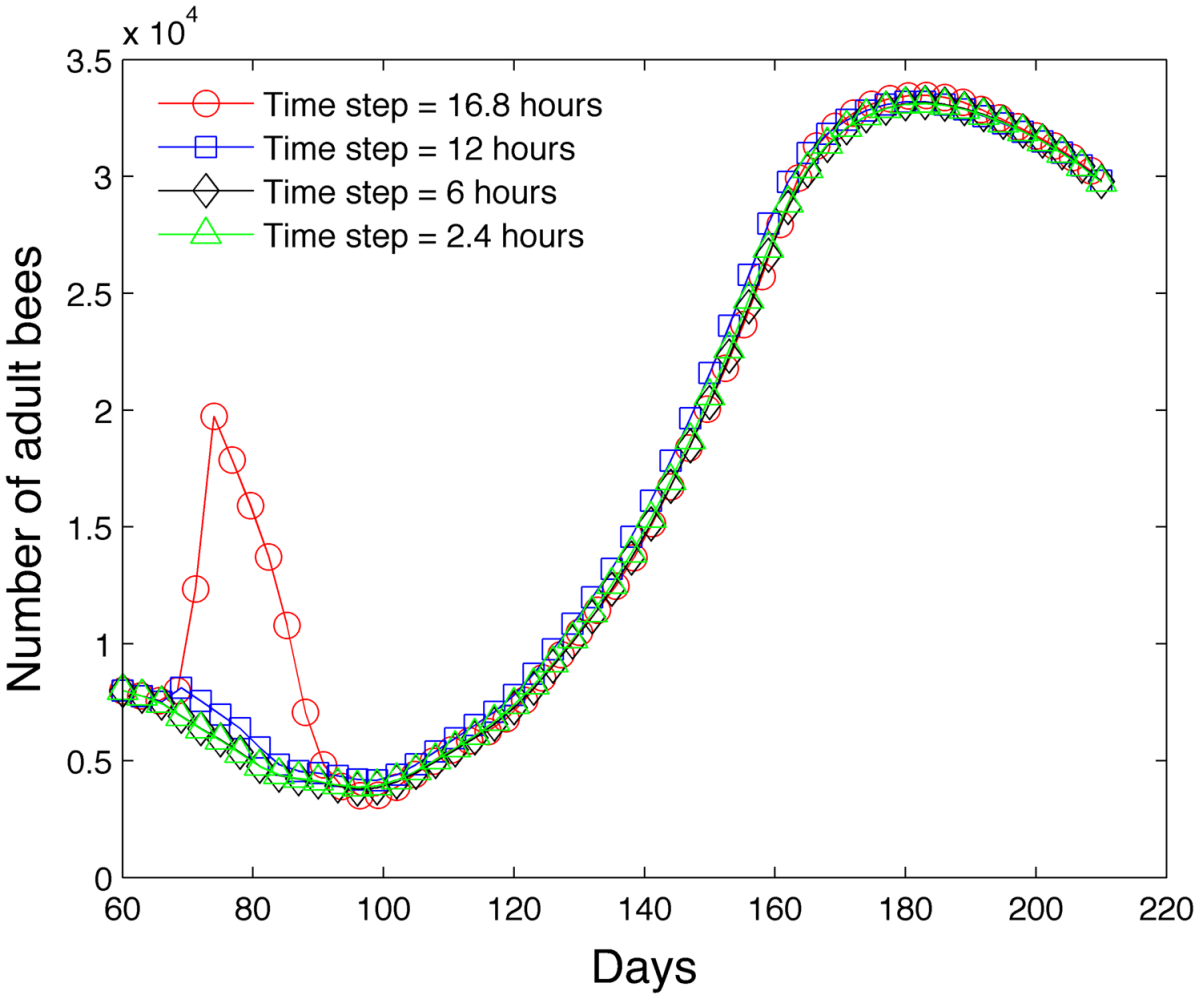
Effect of time step on the accuracy of the model in predicting the number of adult bees.

To make the determination of how small △*t* should be, we perform a convergence study. We note that if *a*_*i*_ = 3, the time step should be at least a third of a day to properly accelerate the maturation of hive bees. A 150 day simulation is performed with four different time steps: 16.8 hours, 12 hours, 6 hours and 2.4 hours. We use the seasonal equation from Schmickl et al. [13] to model the term *s*(*t*) used in (14) and (31),

$$\begin{array}{c} s\left(t\right)=1-max\{\begin{array}{c} 1-\frac{1}{1+x_{1}exp\left(-[2t/x_{2}]\right)}\\ \frac{1}{1+x_{3}exp\left(-[2\left(t-x_{4}\right)/x_{5}]\right)} \end{array} \end{array}$$ (32)

with *x*_1_ = 385, *x*_2_ = 30, *x*_3_ = 36, *x*_4_ = 155, *x*_5_ = 30 and *t* is the day of the year. The egg laying rate is assumed to be *B*_0_ = 1600*s*(*t*) eggs per day. We begin with 8,000 hive bees. Our goal is to determine the time step below which the evolution of the adult bees is independent of the time step. Fig 6 shows the results of the simulations. On the horizontal axis, a value of *t* = 1 refers to January 1st and a value of *t* = 365 refers to December 31st. When the time step is 16.8 hours, the simulation becomes unstable. Below a time step of 6 hours, there no visual difference between the graph and the simulation that uses a time step of 2.4 hours. Therefore we believe that △*t* should be 6 hours or smaller. This is an important observation since many models [11, 12] use a time step of one day, although we acknowledge that their specific equations may not require as restrictive of a time step.

All succeeding simulations we present use a time step of 2.4 hours. Most simulations run under 15 seconds using a MacBook Pro 2.5 GHz processor with 4GB of RAM.

### 2.6 Agreement with steady state

**Fig 7:**
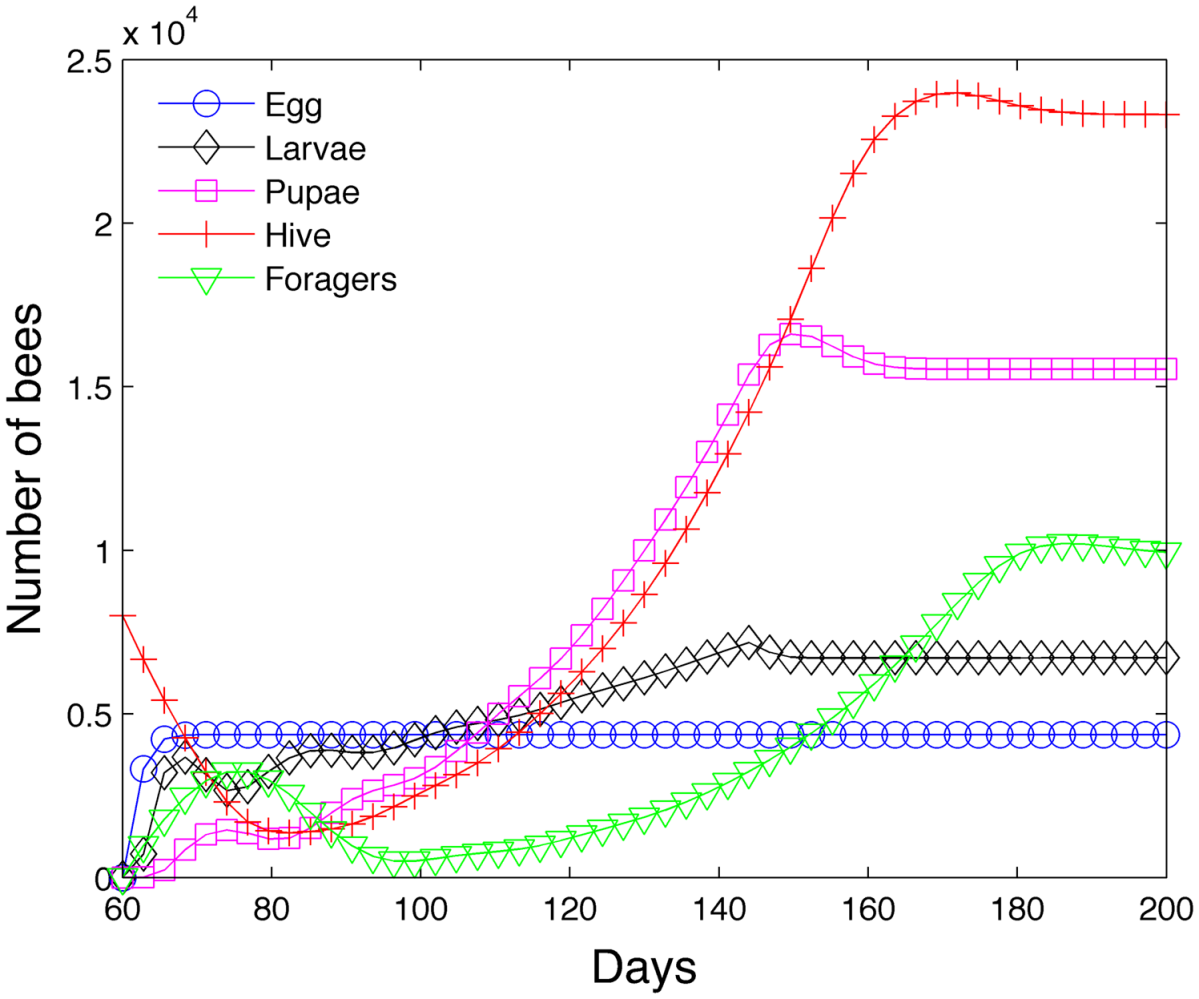
Achievement of steady state in the absence of seasonal effects.

We test the model without any seasonal effects *s*(*t*) = 1 and the pheromone modules deactivated, *a*_*i*_ = 1. We achieve the same steady state numbers as shown in row I of Table 4 and confirm the agreement between the steady state model and the transient model. Fig 7 shows how the bee class numbers stabilize and approach their steady state values.

## 3 Results

**Fig 8:**
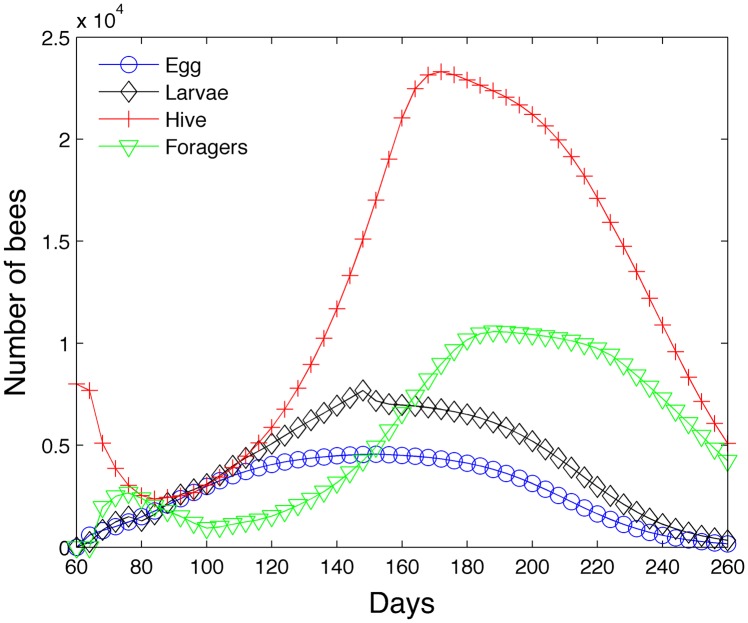
Transient model—200 days.

The model is first run with 2000 grams of food for 200 days with seasonal effects in the northern hemisphere. Fig 8 shows the simulation (egg, larvae, hive and forager bees) run using *B*_0_ = 1600*s*(*t*) starting on day 60 or March 1st. Initially the colony houses only 8000 hive bees. In the first week, the population of hive bees declines while the forager population increases due mainly to aging. Simultaneously the population of larvae begins to increase because eggs are being laid by the queen. Eventually enough larvae mature to offset the declining population of hive bees. However, the dip in the number of hive bees (around day 85) shows up later as a dip in the foraging population (around day 100). The number of eggs peak around day 150 and then began to decline. The number of larvae, hive, and foraging bees also subsequently peak (in that order) and decline.

**Fig 9:**
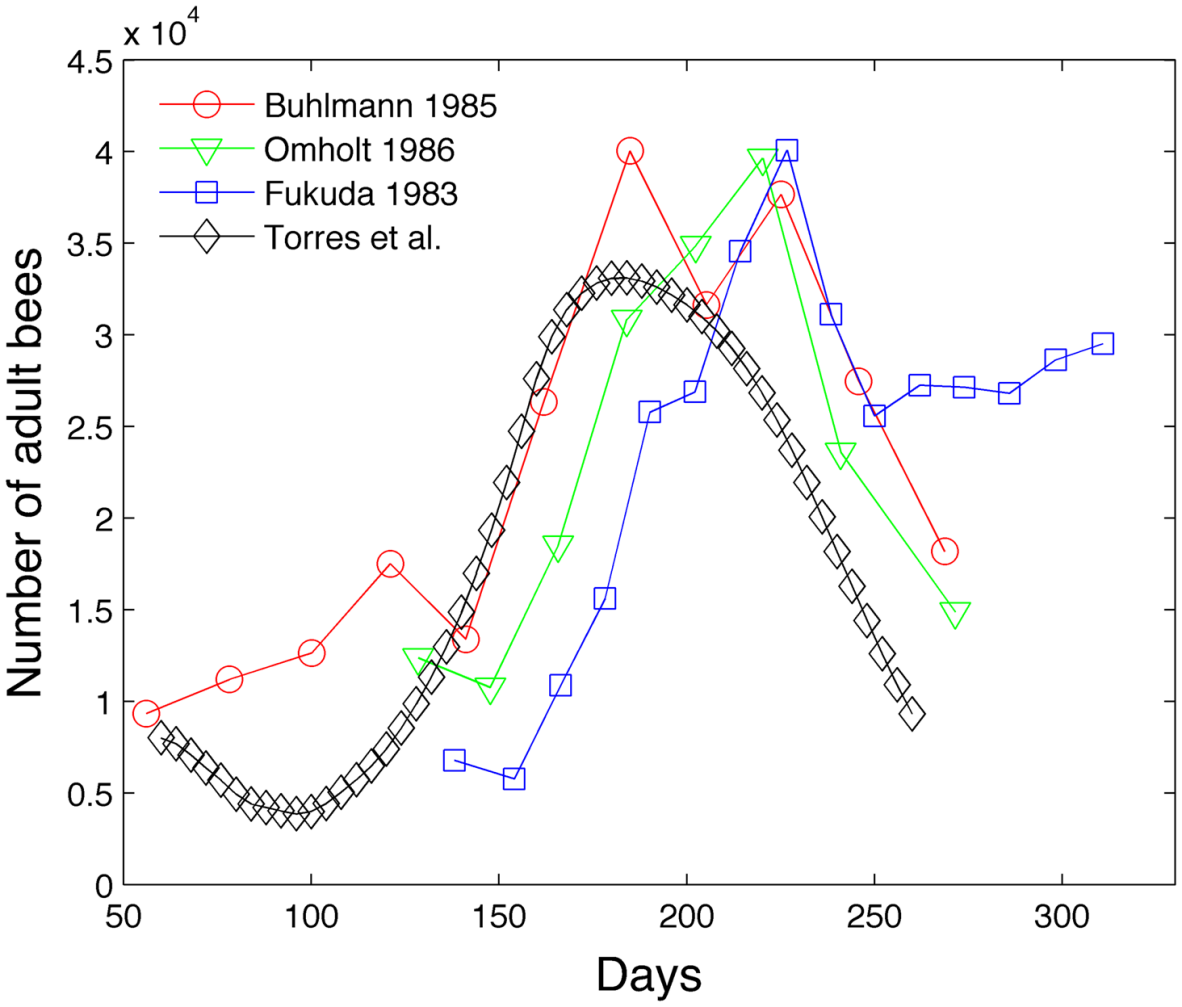
Model comparison of adult bees with experimental data.

**Fig 10:**
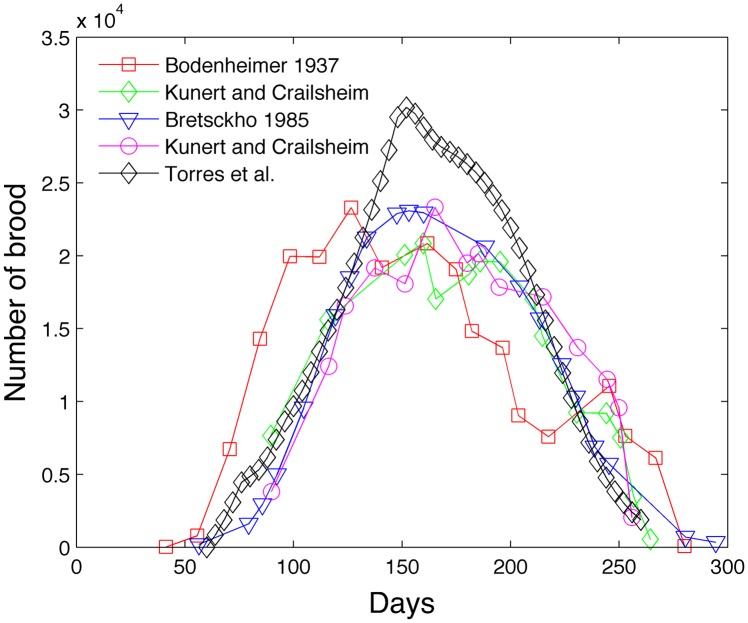
Model comparison of brood with experimental data.

We extract experimental data from Fig 3a and 3b from Schmickl and Crailsheim [13] to construct two figures. Fig 9 compares our model with experimental population of adult bees from sources Omholt [37], Fukuda [38], and Bühlmann [39]. Schmickl and Crailsheim normalize the experimental data because the experimental data was collected for different sizes of honey bee colonies. Fig 10 compares our model with the experimental brood population from sources Bretschko [40], Bodenheimer [41] and Kunert and Crailsheim [42]. Our model colony seems to lie within the range of variability of experimental data, although our brood size peak seems high and our adult bee size peak seems low. Becher et al. [14] also compare their model with the empirical data from [37–39]. They also include brood cell data from Imdorf et al. [43] who show that the number of brood cells peaks between 23,000 to 34,000. We acknowledge that the dynamics of experimental bee populations will depend on the geographical location and length of the foraging season [7].

**Fig 11:**
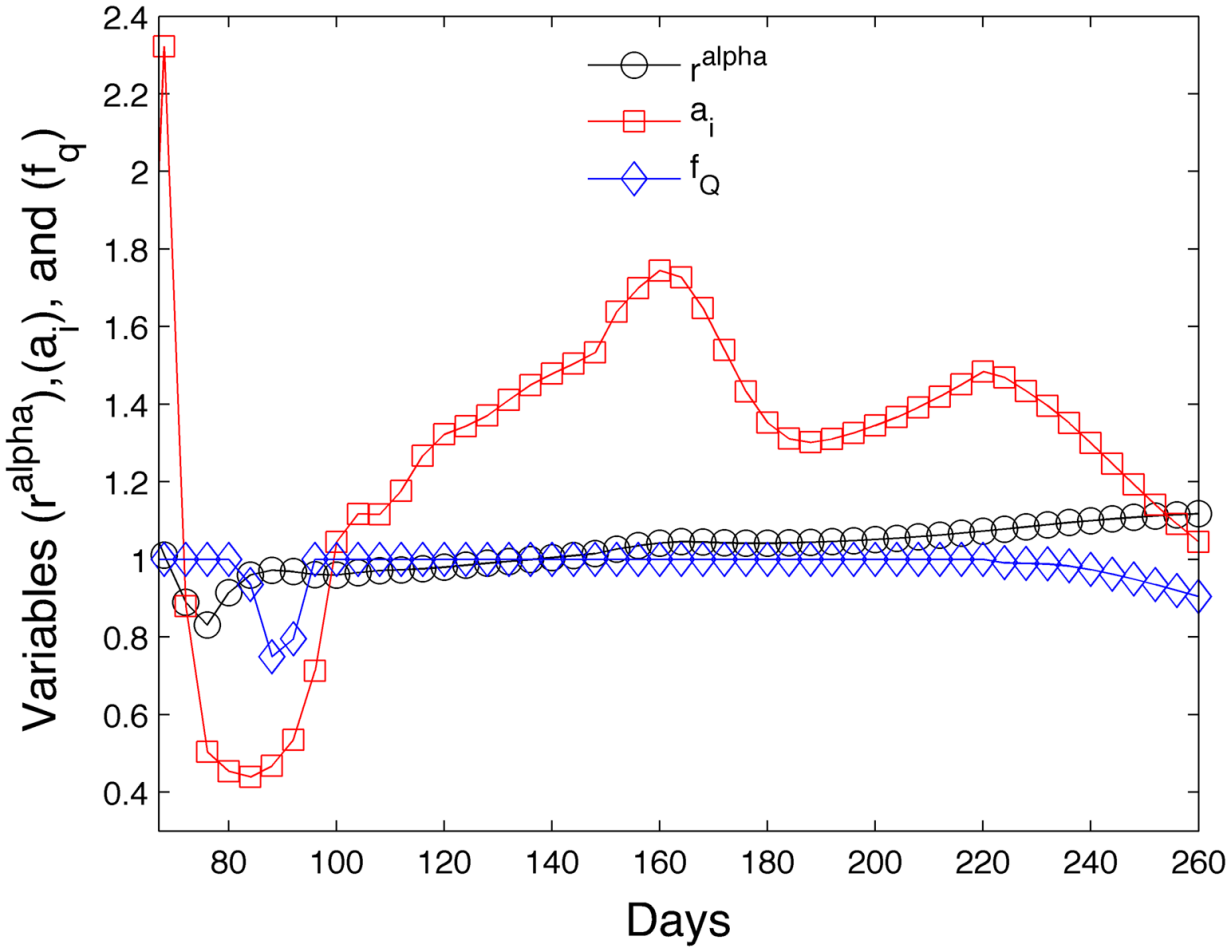
Evolution of variables which affect larvae mortality (*r* ^*α*^), hive bee maturation rate (*a*_*i*_) and foraging rate (*f*_*Q*_).

Fig 11 shows the ratio *r* ^*α*^ from Eq (15), the acceleration term *a*_*i*_ from Eq (22), and the forager rate reduction *f*_*Q*_ from Eq (18) as a function of time for the simulation shown in Fig 8. When *r* ^*α*^ < 1, the larvae in the colony experience increased mortality due to insufficient nurse bees according to (9). When *r* ^*α*^ > 1, the larvae mortality is assumed to be the normal rate from row 1 in Table 3, *m* = 1 − *S* = 1 − .99 = .01. We see that the colony experiences some larvae deaths due to insufficient nurse bees from days 69 to 136. When *a*_*i*_ > 1, pheromones accelerate the development of hive bees. When *a*_*i*_ < 1, pheromones decelerate the development of hive bees to retain more hive bees. Pheromones decelerate the hive bee maturation rate in days 72 to 99. Outside that range, the hive bee maturation rate is accelerated. When *f*_*Q*_ < 1 the foraging food rate *p* is reduced. We see a reduction in the foraging rate in days 80 through 96 and after day 220.

**Fig 12:**
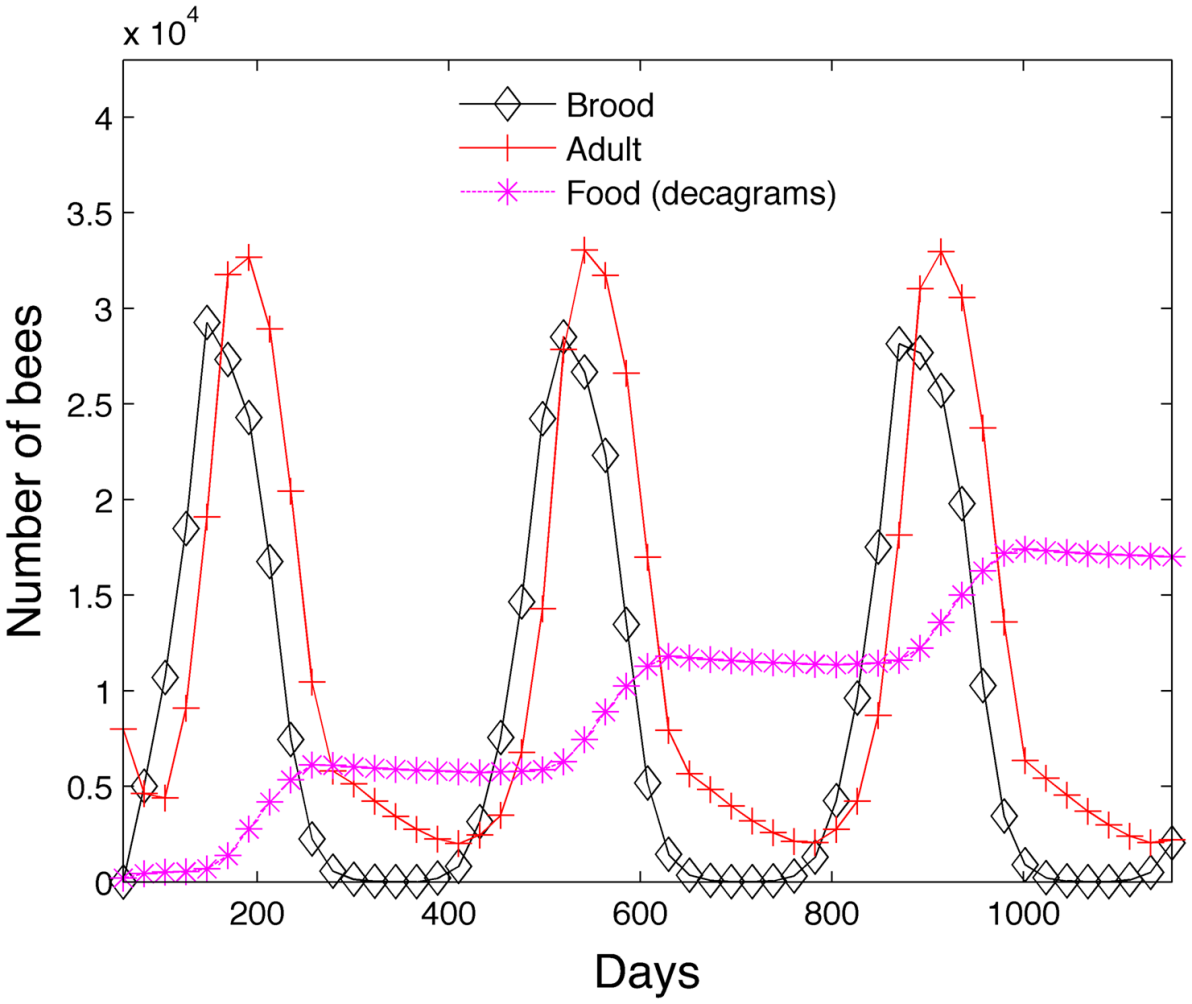
Transient model—Three years.

Fig 12 shows the simulation with the same initial conditions run over 3 years. Adult bees (hive + foragers) and brood (egg + larvae + pupae) are shown. During the winter phase (September 17th—March 5th) we reduce the mortality of the hive bee *m*_*i*_ = .01 and assume all hive bees stay hive bees even after *n*_*hive*_ = 21 days. The queen also ceases to produce eggs. We note that the colony is producing much more food than it requires. The food reserves (shown in decagrams) show a rapid increase during the summer months and a very gradual decrease during the winter months. In both the 200 day and three year simulations, no bees die due to insufficient food if the colony begins with 2000 grams of food.

### 3.1 Sensitivity studies

In the steady state and transient models, some parameters remain unknown or difficult to estimate. Therefore, we perform sensitivity studies to assess the impact of different levels of a specific parameter on the bee colony. We assume the same initial conditions as the 200 day and 3 year simulations (8000 hive bees on day 60).

#### 3.1.1 Impact of insufficient nurse bees

**Fig 13:**
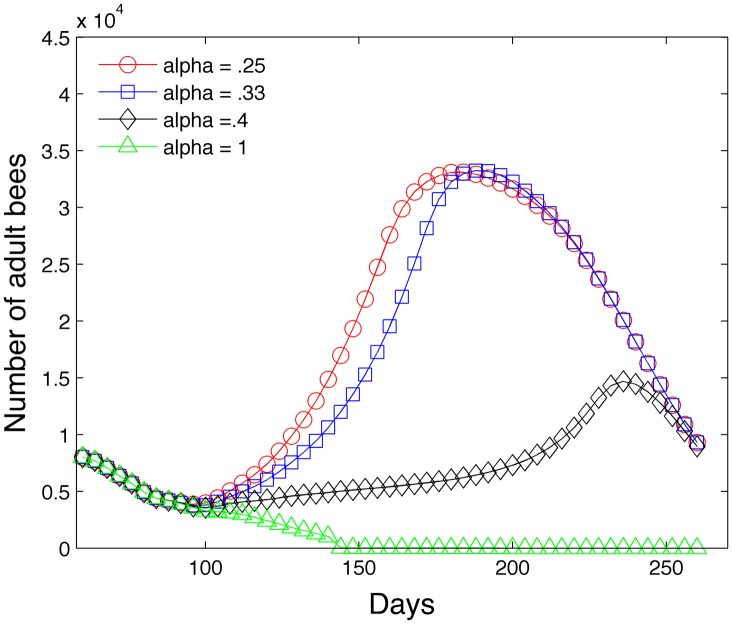
Sensitivity study with different levels of *α*.

The first study varies the parameter *α* in Eq (9) which regulates the impact of insufficient nurse bees on the larvae population. Fig 13 shows the effect of the parameter *α* in a one year simulation of a bee colony. We observe that too large an *α* = 1 can cause the colony to fail and large values of *α* = .4 can have a noticeable negative impact. Recall that large *α* values produce higher larvae mortality rates in the absence of sufficient nurse bees.

#### 3.1.2 Impact of the healthy ratio of hive to forager bees

**Fig 14:**
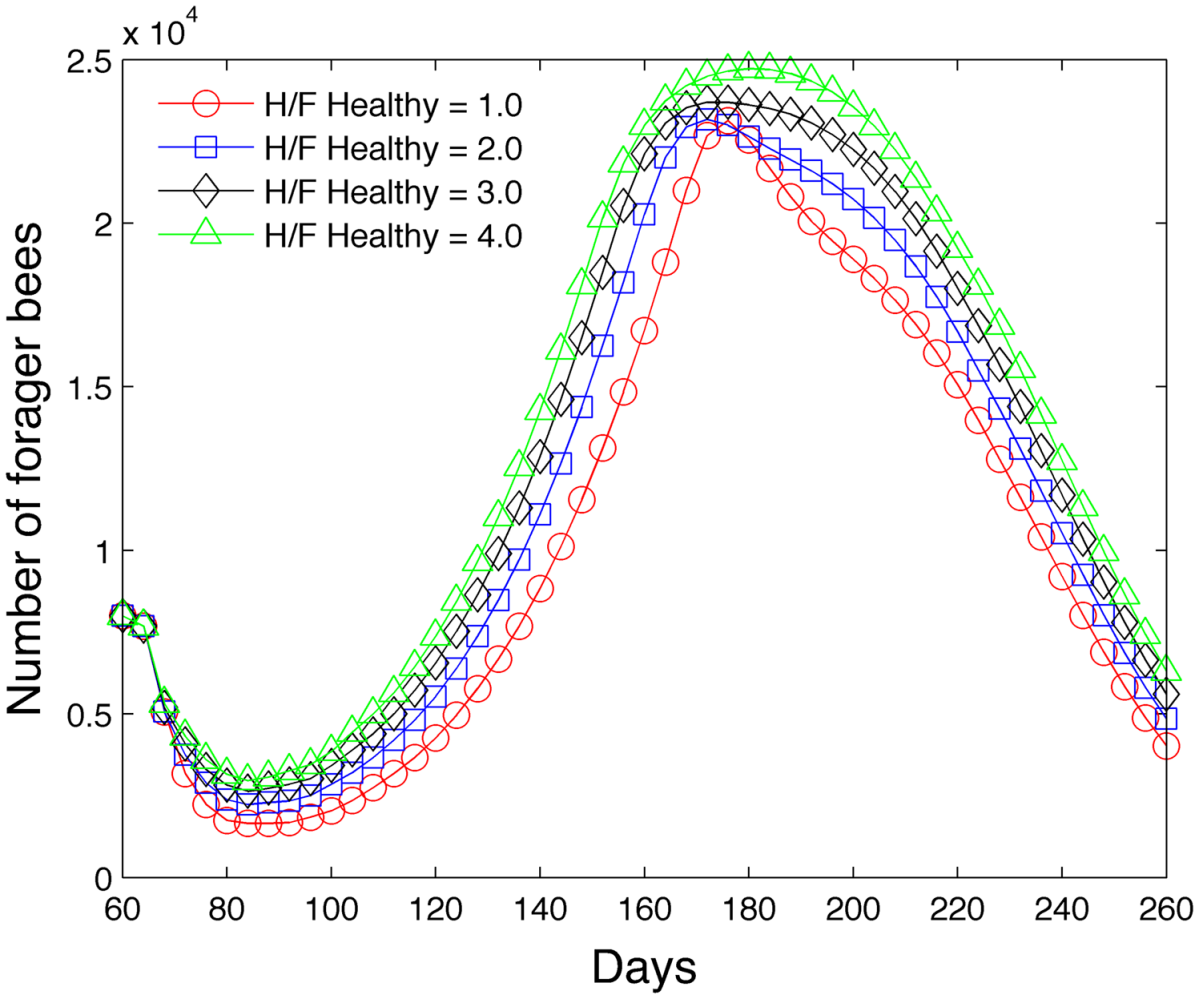
Effect of ${\left(R_{F}^{H}\right)}_{healthy}$ on hive population.

**Fig 15:**
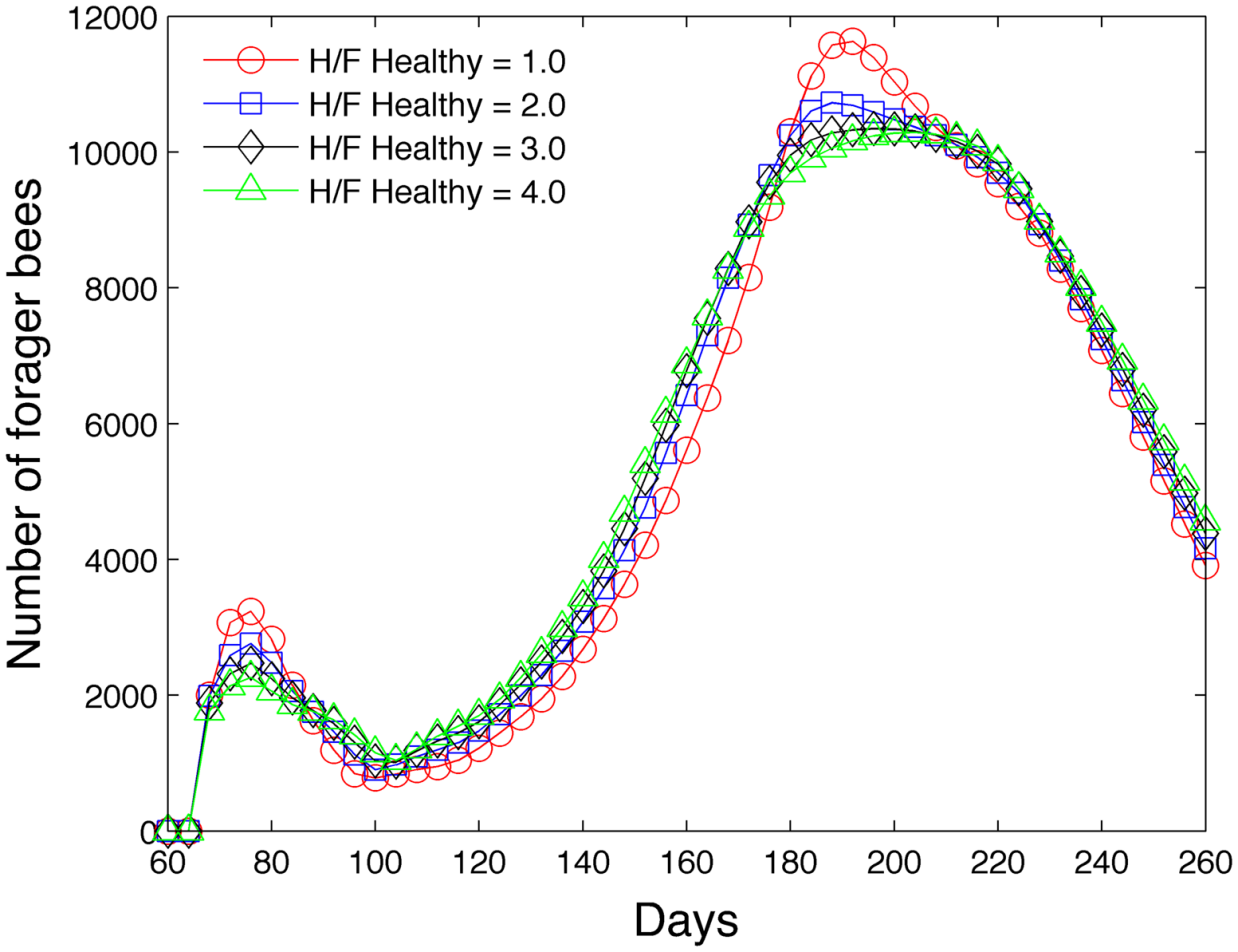
Effect of ${\left(R_{F}^{H}\right)}_{healthy}$ on forager population.

The second study varies the parameter ${\left(R_{F}^{H}\right)}_{healthy}$ in Eq (24) and determines its effect on the hive and forager population. Fig 14 shows that as ${\left(R_{F}^{H}\right)}_{healthy}$ increases, the hive population increases due to the impact of the pheromone ethyl oleate and its bias toward increasing the numbers of hive bees relative to foraging bees. Similarly as expected, Fig 15 shows that as ${\left(R_{F}^{H}\right)}_{healthy}$ increases, the forager population tends to decrease (on days near 76 and 200) due to the decelerated hive maturation rate. However, we note that the overall lifespan of a bee that matures early is less than a bee that matures late because of the high mortality rate in the forager caste [44]. For this reason, the number of foragers is actually less at low ${\left(R_{F}^{H}\right)}_{healthy}$ ratios at certain times during the year.

#### 3.1.3 Impact of summer duration

**Fig 16:**
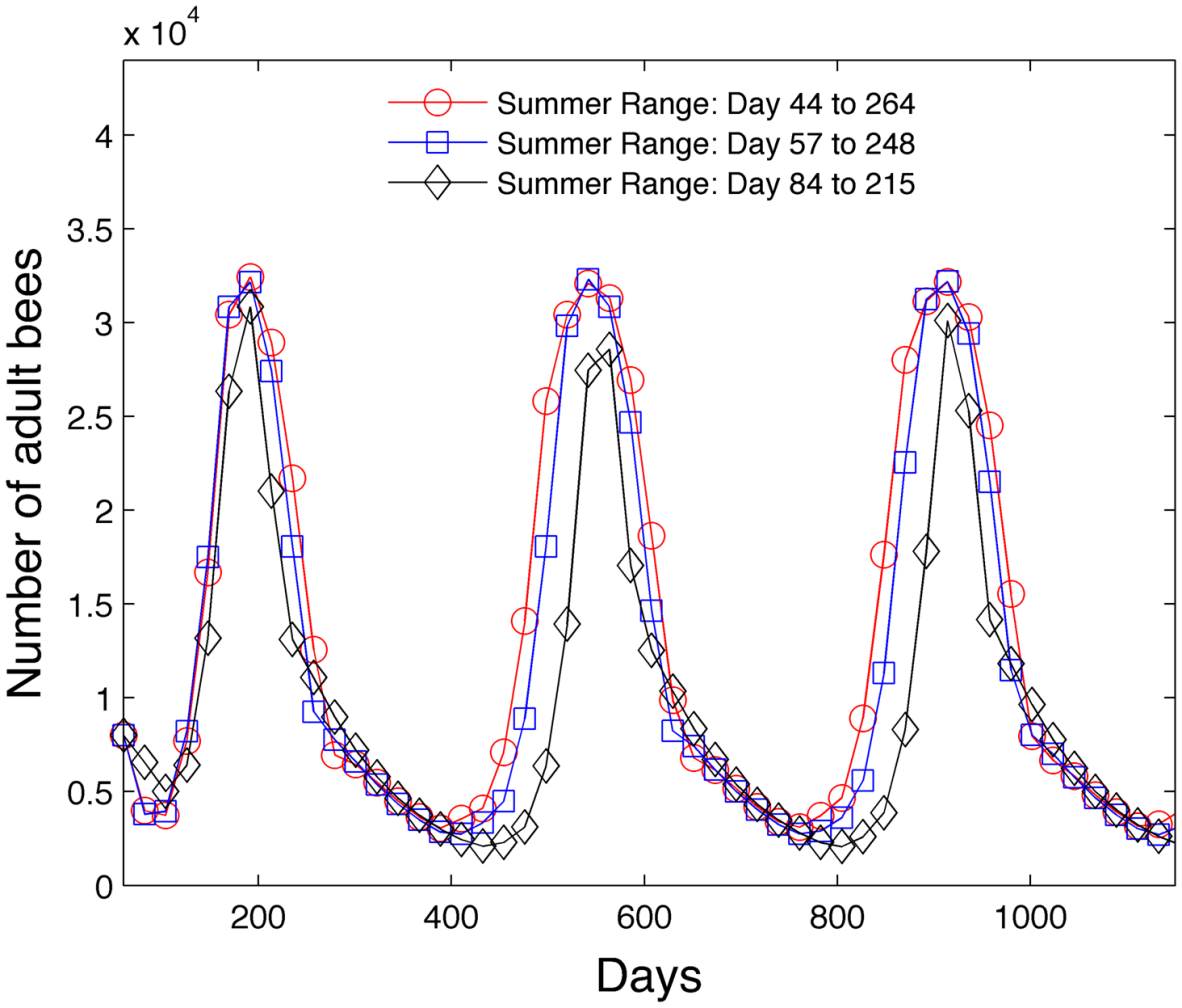
Effect of length of summer on adult bee population.

The third study varies the length of the summer by modifying Eq (32) which determines the egg laying rate $B_{0}=1600\tilde{s}(t)$. We use a similar form

$$\begin{array}{c} \tilde{s}\left(t\right)=1-\{\begin{array}{cc} \frac{1}{1+x_{3}exp\left([2\left(t-{\tilde{x}}_{4}\right)/\left({\tilde{x}}_{5}+5\right)]\right)} & t<150\\ \frac{1}{1+x_{3}exp\left(-[2\left(t-{\tilde{x}}_{4}\right)/{\tilde{x}}_{5}]\right)} & t\geq 150 \end{array} \end{array}$$ (33)

but one which is easier to manipulate through one parameter ${\tilde{x}}_{5}$. The parameters are assumed to be *x*_3_ = 36, ${\tilde{x}}_{4}=150$, ${\tilde{x}}_{5}=\{35,30,20\}$. The onset of summer is assumed to occur when $\tilde{s}(t)$ reaches a value of .15 during the spring and decreases to .05 during the fall. The winter days outside of summer determine when the hive bees cease to develop into foragers and experience a reduced mortality of .01. Note that $\tilde{s}(t)$ has a maximum value of .973 while *s*(*t*) has a maximum of 1. The length of the summer occurs from days 44 to 264 when ${\tilde{x}}_{5}=35$, days 57 to 248 when ${\tilde{x}}_{5}=30$, and days 84 to 215 when ${\tilde{x}}_{5}=20$. Fig 16 shows that the shortened summer 84–215 reduces the peak adult population. However, summer day ranges 44–264 and 57–248 have similar adult bee peak values despite the fact that the summer is 29 days shorter for the summer day range 57–248 simulation.

#### 3.1.4 Impact of foraging rate

**Fig 17:**
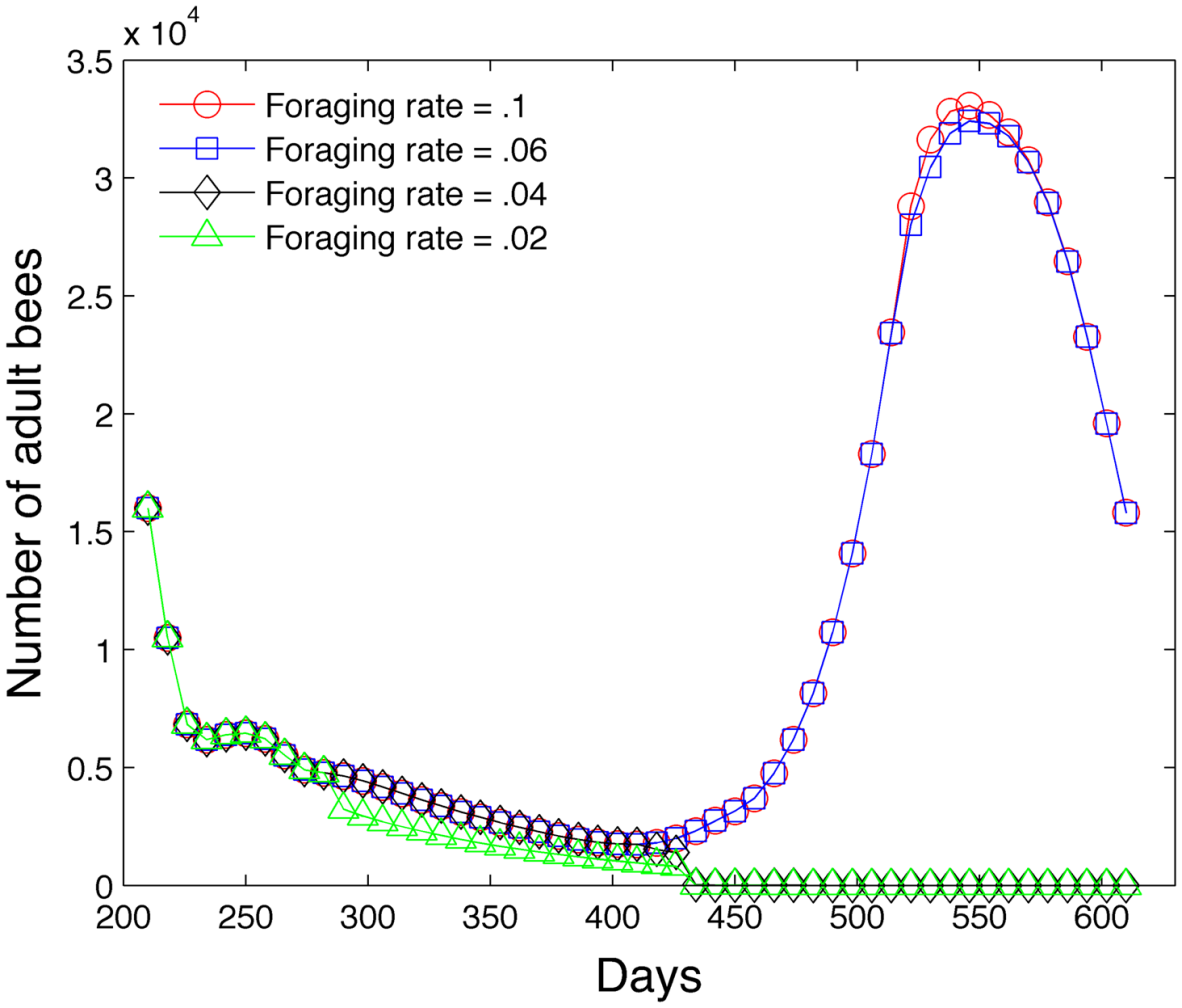
Effect of different levels of the foraging rate *p* on adult bee population.

The next study varies the daily foraging rate *p* in Eq (19) and determines its effect on the adult bee population. The calculation begins on day 210 with 10,400 hive bees, 5,600 foraging bees and 2 kg of food. All previous simulations do not invoke the food scarcity algorithm because sufficient food is provided and obtained by the colony. However, this simulation specifically stresses the colony through food scarcity by reducing the foraging rate. Fig 17 shows that the colony collapses when *p* = .04 *g*/(*day*⋅*bee*) and *p* = .02 *g*/(*day*⋅*bee*) due to insufficient food at day 430 or in early March. The foraging rate curve *p* = .04 *g*/(*day*⋅*bee*) is difficult to discern but follows the curve for *p* = .06 *g*/(*day*⋅*bee*) and declines and diverges at 420 days. A foraging rate of *p* = .06 *g*/(*day*⋅*bee*) is sufficient to sustain the colony.

#### 3.1.5 Impact of pheromones and cannibalism

**Fig 18:**
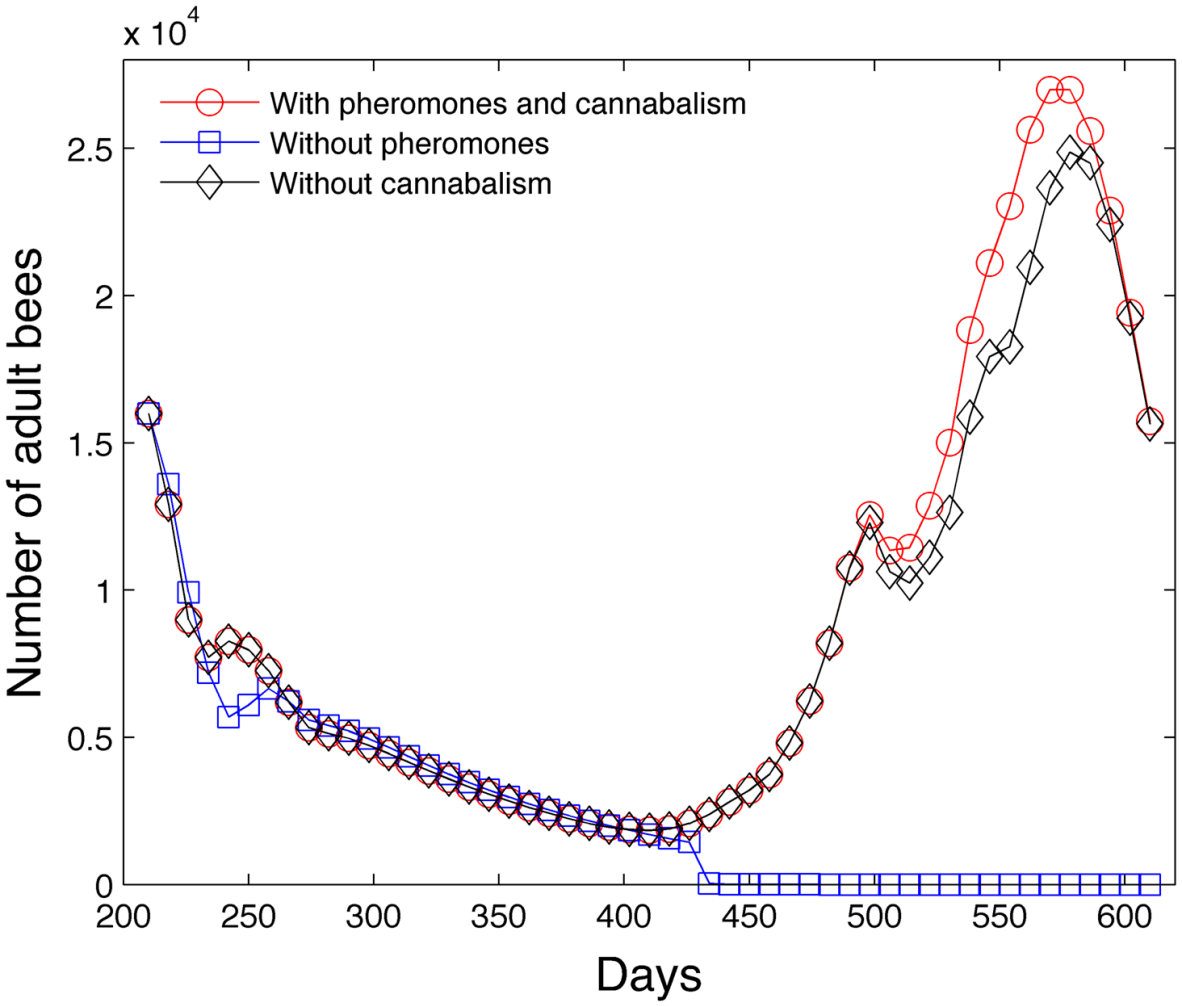
Colony collapses without pheromones or cannibalism with forager rate *p* = .055 *g*/(*day*⋅*bee*).

Fig 18 shows that pheromones and cannibalism help with the survival of the colony under low forager rates *p* = .055 *g*/(*day*⋅*bee*). Without the use of pheromones, the colony collapses around day 430. The colony population growth is slightly less when the colony abstains from cannibalism.

## 4 Conclusion

We have designed a steady state model and a transient model of honey bee populations. The steady state model is used to demonstrate that the honey bee colony is susceptible to mortality rates in the pupae, larvae and hive castes. We also demonstrate how brood pheromone and ethyl oleate pheromone aid in the survival of the colony by allowing the colony to be self-sufficient in regards to food under higher forager mortality rates.

Our transient model accounts for seasonal effects and time variations within the population and is developed using differential equations. Larvae mortality is increased in the absence of sufficient hive bees. Pheromones are accounted for by accelerating or decelerating the development of hive bees. Food scarcity is accounted for by decreasing the survival rates of bee castes. A 200 day and a three year simulation are performed and our model is compared with experimental results. In addition, sensitivity studies are conducted which show the effect of varying parameters which regulate larvae mortality, healthy ratios of hive to forager bees, summer duration and food foraging rates. The transient model shows that pheromones and cannibalism aid in the survival of the colony under low food foraging rates.

Improvements in the model depend on improving the accuracy of the parameters. Accurate healthy ratios of hive to larvae bees, hive to foraging bees, and processing to foraging bees are important components of the model since they influence the survival rate of larvae, the impact of pheromones, and the food collection rate. An accurate determination of the egg laying rate and forager lifespan throughout the nonwinter seasons are other parameters that could benefit from more experimental data.
